# Single- and Two-Electron Reduction of Nitroaromatic Compounds by Flavoenzymes: Mechanisms and Implications for Cytotoxicity

**DOI:** 10.3390/ijms22168534

**Published:** 2021-08-08

**Authors:** Narimantas Čėnas, Aušra Nemeikaitė-Čėnienė, Lidija Kosychova

**Affiliations:** 1 Institute of Biochemistry of Vilnius University, Saulėtekio 7, LT-10257 Vilnius, Lithuania; lidija.kosychova@bchi.vu.lt; 2 State Research Institute Center for Innovative Medicine, Santariškių St. 5, LT-08406 Vilnius, Lithuania; ausra.ceniene@imcentras.lt

**Keywords:** nitroaromatic compounds, flavoenzymes, cytotoxicity, oxidative stress, bioreductive activation

## Abstract

Nitroaromatic compounds (ArNO_2_) maintain their importance in relation to industrial processes, environmental pollution, and pharmaceutical application. The manifestation of toxicity/therapeutic action of nitroaromatics may involve their single- or two-electron reduction performed by various flavoenzymes and/or their physiological redox partners, metalloproteins. The pivotal and still incompletely resolved questions in this area are the identification and characterization of the specific enzymes that are involved in the bioreduction of ArNO_2_ and the establishment of their contribution to cytotoxic/therapeutic action of nitroaromatics. This review addresses the following topics: (i) the intrinsic redox properties of ArNO_2_, in particular, the energetics of their single- and two-electron reduction in aqueous medium; (ii) the mechanisms and structure-activity relationships of reduction in ArNO_2_ by flavoenzymes of different groups, dehydrogenases-electrontransferases (NADPH:cytochrome P-450 reductase, ferredoxin:NADP(H) oxidoreductase and their analogs), mammalian NAD(P)H:quinone oxidoreductase, bacterial nitroreductases, and disulfide reductases of different origin (glutathione, trypanothione, and thioredoxin reductases, lipoamide dehydrogenase), and (iii) the relationships between the enzymatic reactivity of compounds and their activity in mammalian cells, bacteria, and parasites.

## 1. Introduction

Over the decades, nitroaromatic compounds (ArNO_2_) maintain their importance in relation to industrial processes, environmental pollution, and pharmaceutical application. Current estimates have their production, that is, the synthesis of pigments, polymers, pesticides, explosives, or pharmaceuticals, up to 10^8^ tons per year ([1,2,3,4,5,6], and references therein). Because of contamination of groundwater and soil at military and industrial sites by ArNO_2_ that exhibit toxic, mutagenic, and cancerogenic activities, there has been a significant increase in research to understand and apply biological processes for their degradation.

**Figure 1:**
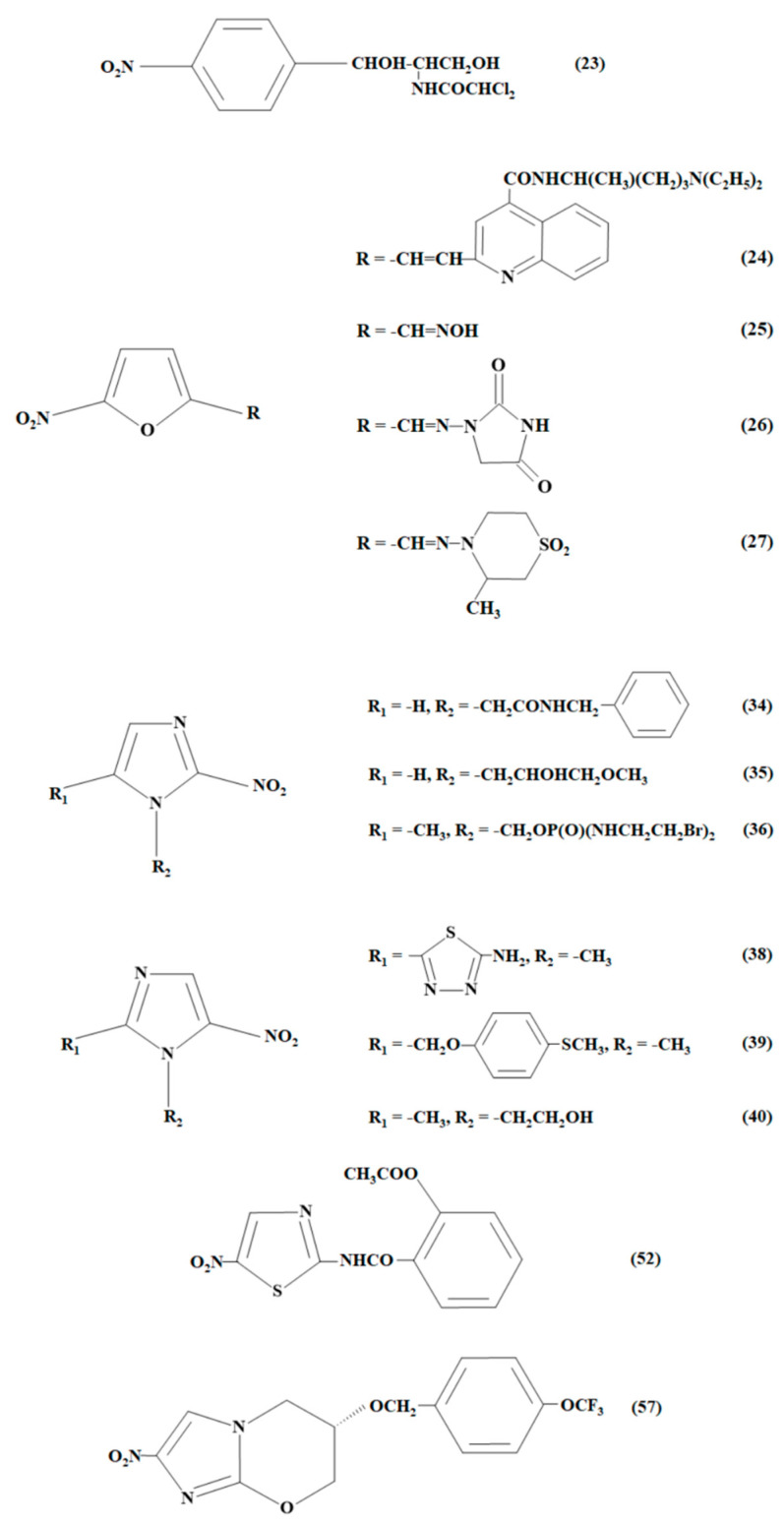
Formulas of nitroaromatic antibacterial and antiparasitic agents: chloramphenicol (**23**), chinifur (**24**), nifuroxime (**25**), nitrofurantoin (**26**), nifurtimox (**27**), benznidazole (**34**), misonidazole (**35**), TH-302 (**36**), megazol (**38**), fexinidazole (**39**), metronidazole (**40**), nitazoxanide (**52**), and PA-824 (**57**). The numbers of compounds correspond to those in Table A1 (Appendix A).

**Figure 2:**
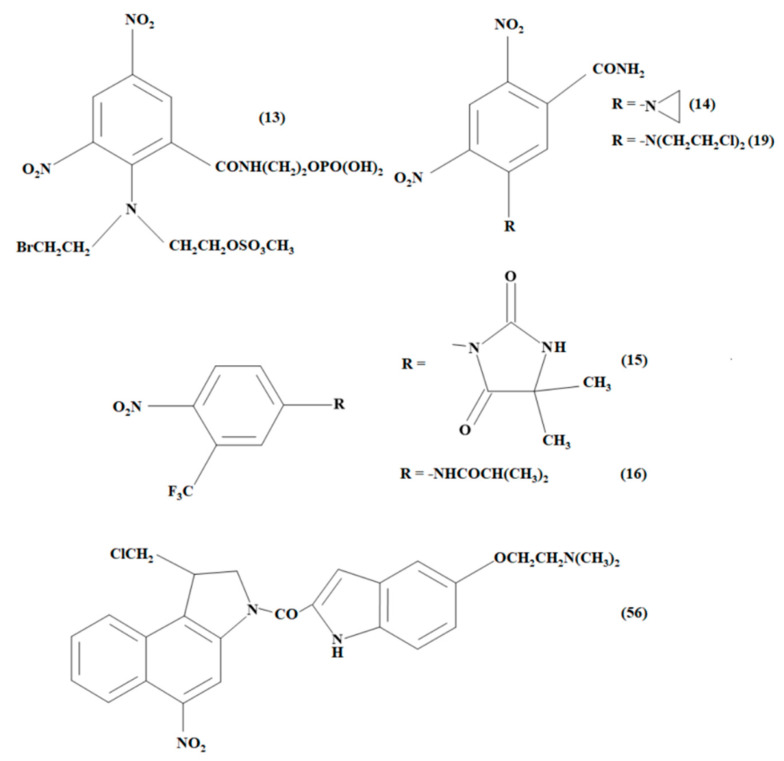
Formulas of nitroaromatic anticancer agents: PR-104 (**13**), CB-1954 (**14**), SN-3862 (**19**), nilutamide (**15**), flutamide (**16**), and a representative of nitroCBIs, 1-(chloromethyl)-3-(5-(2-(dimethylamino-ethoxy)indol-2-carbonyl)-5-nitro-1,2-dihydro-3*H*-benzo[*e*]-indole (**56**). The numbers of compounds correspond to those in Table A1 (Appendix A).

On the other hand, the electron-attracting ability and redox activity make the nitro group a versatile and unique group in medicinal chemistry. Nitroaromatic compounds have a long history of use as antibacterial and antiparasitic drugs and their application as radiosensitizers and hypoxia-selective anticancer agents ([6], and references therein) (Figure 1 and Figure 2). The resurgence of interest in their use is caused by the reevaluation of the problems with their mutagenicity and the new potential fields of their application, e.g., the treatment of oxic tumors, including the development of antibody- or gene-directed therapies employing bacterial nitroreductases [7,8].

**Figure 3:**
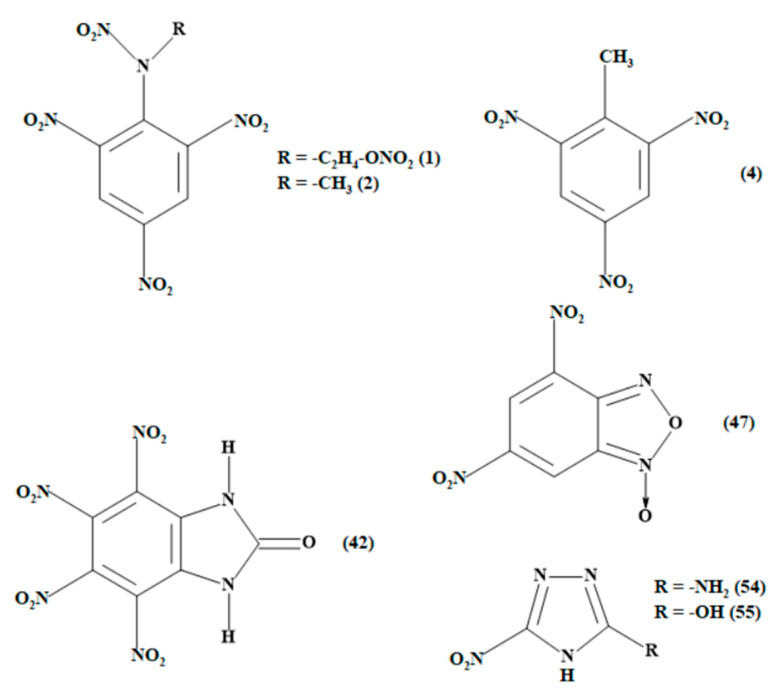
Formulas of nitroaromatic explosives: pentryl (**1**), tetryl (**2**), 2,4,6-trinitrotoluene (TNT) (**4**), 4,5,6,7-tetranitrobenzimidazolone (**42**), 4,6-dintrobenzofuroxane (**47**), ANTA (**54**), and NTO (**55**). The numbers of compounds correspond to those in Table A1 (Appendix A).

Importantly, both the biodegradation of environmental pollutants such as explosives such as 2,4,6-trinitrotoluene (TNT) (**4**) or 2,4,6-trinitrophenyl-*N*-methylnitramine (tetryl) (**2**) (Figure 3) and the manifestation of toxicity/therapeutic action of nitroaromatic drugs (Figure 1 and Figure 2) may involve similar initial steps, single- or two-electron reduction in ArNO_2_ performed by various flavoenzymes and/or their physiological redox partners, metalloproteins. However, in spite of the rapidly increasing amount of information in this area, the pivotal and still incompletely resolved questions are the identification of the specific enzymes that are involved in the bioreduction of nitroaromatics, the characterization of their reaction mechanisms, and the establishment of their contribution to cytotoxic/therapeutic action of ArNO_2_.

This review, although it is not meant to be exhaustive, addresses the above problems with special emphasis on the characterization of flavoenzymes performing single- and two-electron reduction in nitroaromatics, the mechanisms and structure-activity relationships of reactions, and the relationships between the reactivity of compounds and their activity in biological systems.

## 2. Redox Properties of Nitroaromatic Compounds and Their Reduction Products

The quantitative characterization of intrinsic redox properties of nitroaromatic compounds is instrumental in the analysis of their enzymatic reduction mechanisms. In this part, we attempt to address the energetics of single- and two-electron reduction in ArNO_2_ in aqueous medium and some relevant properties of their reduction products. Another important mechanism of their reduction, the formation of Meisenheimer-type hydride adducts [9], is beyond the scope of this review because it is more relevant to the biodegradation of ArNO_2_ rather than their cytotoxicity.

ArNO_2_ can be reduced by multistep net six-electron transfer into corresponding amines (ArNH_2_) with the formation of anion-radical (ArNO_2_^−^), nitroso (ArNO), and hydroxylamine (ArNHOH) intermediates. In this aspect, the best-characterized is the energetics of first electron transfer, described by a midpoint redox potential of the ArNO_2_/ArNO_2_^−^· couple (*E*^1^, or *E*^1^_7_ at pH 7.0). Due to the instability of free radicals in aqueous media, the *E*^1^_7_ values of nitroaromatics (Table A1, Appendix A) are usually obtained from anaerobic pulse radiolysis experiments [10,11,12,13,14,15,16,17,18,19,20,21,22,23,24]. The range of *E*^1^_7_ values of ArNO_2_ with biomedical interest is from −0.6 V to −0.2 V. Further in the text, all the potentials will be given with respect to NHE. For most important groups of compounds, *E*^1^_7_ decreases in the order nitropyridines > nitrofurans ≥ nitrothiophenes > nitrobenzenes > nitroimidazoles (Table A1). For the series of homologous compounds, their *E*^1^_7_ values may be roughly correlated with the σ values of their substituents. In addition, the value of *E*^1^_7_ decreases if a nitro group loses a coplanarity with the aromatic system due to sterical hindrances. The *p*K_a_ values of ArNO_2_^−^·span from 2.0 to 3.0 (nitrobenzenes, nitrofurans) [25,26] to 5.7–6.1 (nitroimidazoles) [27]. In terms of an “outer-sphere” electron transfer mechanism ([28,29,30], and Appendix B), the electron self-exchange rate constants of ArNO_2_ are ~10^6^ M^−1^s^−1^ [25,26].

Alternatively, the values of *E*^1^ of the number of nitroaromatics were determined by cyclic voltammetry. Typically, the electrochemical reduction in ArNO_2_ in aqueous media proceeds irreversibly with the formation of ArNHOH. However, this process takes place in two steps, with the pH-independent transfer of the first electron and pH-dependent transfer of three electrons:

ArNO_2_ + e^−^ → ArNO_2_^−^, (1)

ArNO_2_^−^ + 3e^−^ + 4H^+^ → ArNHOH + H_2_O. (2)

At pH 11–12, the redox potential of a second step may become more negative than the potential of ArNO_2_/ArNO_2_^−^· couple. In this case, a separate reversible process of single-electron transfer at *E*_m_ = *E*^1^_7_ is observed in cyclic voltammetry ([31], and references therein).

There is some interest in the prediction of *E*^1^_7_ of ArNO_2_ from quantum mechanical calculations or the use of substitute descriptors such as the electron affinities of ArNO_2_ or the heats of formation (∆Hf) of ArNO_2_^−^. However, the calculations in vacuo frequently do not provide reliable predicted *E*^1^_7_ values due to the large data scattering or may be confined only to a series of homologous compounds [32,33,34,35]. Some improvement may be expected upon the introduction of solvent matrix effects into calculations [36]. On the other hand, the *E*^1^_7_ values of ArNO_2_ may be predicted from linear log (rate constant) vs. *E*^1^_7_ relationships in single-electron reduction in nitroaromatics by flavoenzymes dehydrogenases-electrontransferases or their redox partners, FeS proteins [11,12,13]. The use of the geometric average of rate constants obtained in several enzymatic systems improves the prediction accuracy. The calculated reduction potentials (*E*^1^_7(calc.)_, Table A1, Appendix A) deviate from the experimental ones by no more than 40 mV (standard deviation, ± 18 mV) and thus should be considered as realistic. Importantly, this approach may be applied for groups of structurally diverse ArNO_2_.

The reoxidation of ArNO_2_^−^·by O_2_ and their dismutation are among the most important factors influencing their cytotoxicity. The oxidation of anion-radicals is accompanied by the formation of superoxide (O_2_^−^) and subsequently, H_2_O_2_:

ArNO_2_^−^·+ O_2_ → ArNO_2_ + O_2_^−^, (3)

2O_2_^−^ + 2H^+^ → H_2_O_2_ + O_2_ (4)

The latter further form cytotoxic hydroxyl radical (OH·) in transition metal-catalyzed Fenton reaction. The rate constants of ArNO_2_^−^ oxidation by O_2_ decrease with an increase in their *E*^1^_7_, for example, 7.7 × 10^6^ M^−1^s^−1^ (nitrobenzene), 1.4 × 10^6^ M^−1^s^−1^ (*p*-nitroacetophenone), 2.5 × 10^5^ M^−1^s^−1^ (nitrofurantoin), 1.5 × 10^5^ M^−1^s^−1^ (nifuroxime) [25,26]. During the single-electron reduction in ArNO_2_ by NAD(P)H-oxidizing flavoenzymes, the reactions (3,4) are responsible for typical redox cycling events, oxidation of significant excess NAD(P)H over ArNO_2_, the stoichiometric to NAD(P)H consumption of O_2_, and superoxide dismutase-sensitive reduction in added cytochrome *c*. The dismutation of nitro anion-radicals yields the nitroso compounds:

2ArNO_2_^−^ + 2H^+^ → ArNO_2_ + ArNO + H_2_O. (5)

The dismutation rate constants (2*k*_d_) are structure-sensitive. For the radicals of *o*-, *m*-, and *p*-dinitrobenzenes, they are equal to 2.4 × 10^6^ M^−1^s^−1^, 8.0 × 10^6^ M^−1^s^−1^, and 3.3 × 10^8^ M^−1^s^−1^, respectively, whereas the radicals of nitroimidazoles and nitrofurans are more stable (2*k*_d_ = 10^4^ ÷ 10^5^ M^−1^s^−1^ [25,26,27]). The competition between the dismutation of ArNO_2_^−^ and their reoxidation by O_2_ is responsible for the formation of a fraction of stable reduction products under partial aerobic conditions [37].

Finally, ArNO_2_^−^ possessing substituents with potential leaving groups may undergo fragmentation, which competes with their reoxidation by O_2_ (Equation (6)). This approach is used in the development of hypoxia-selective antitumour agents such as TH-302 (**36**) [20].

O_2_N-ArCH_2_-N^+^(CH_3_)(CH_2_CH_2_Cl)_2_ + e^−^ → [-O_2_N-ArCH_2_-N^+^(CH_3_)(CH_2_CH_2_Cl)_2_] → → O_2_N-ArCH_2_· + CH_3_-N(CH_2_CH_2_Cl)_2_. (6)

The redox properties of ArNO_2_ multielectron reduction products are insufficiently characterized in quantitative terms. In aqueous medium, ArNO_2_ are electrochemically reduced into ArNHOH directly, bypassing ArNO (Equations (1) and (2)). On the other hand, the use of mixed ethanol-aqueous solution with pH 1.0–4.0 enabled the detection of reduction intermediate dihydroxylamine (ArN(OH)_2_), which further undergoes dehydration [38]:

ArNO_2_ + 2e^−^ + 2H^+^ → ArN(OH)_2_ → ArNO + H_2_O. (7)

However, the voltammetric characteristics of this reaction could not be extrapolated into aqueous medium with pH 7.0. Following this approach, it was assumed that the rate-limiting step of enzymatic two-electron reduction in ArNO_2_ is a net hydride transfer with the formation of ArN(OH)O^−^ [39]. The calculated heats of formation (∆Hf(ArN(OH)O^−^) increase with the electron-accepting potency of substituents and roughly correlate with ∆Hf(ArNO_2_^−^) and *E*^1^_7_ of nitroaromatics.

In aqueous medium, nitrosobenzene is reversibly electrochemically reduced into phenylhydroxylamine at *E*^0^_7_ = 0.184 V [40]. It is suggested that the intermediate free radical ArNOH· is unstable, and that the redox potentials of first and second electron transfer are separated by −0.5 V. Because ArNO are more powerful oxidants than ArNO_2_, they can be directly reduced by NAD(P)H, GSH, ascorbate, and other reductants. For example, nitrosobenzene is reduced by NADPH and ascorbate with *k* = 124 M^−1^s^−1^ and 2.8 × 10^3^ M^−1^s^−1^, respectively [41,42]. The reactivity of nitrosobenzenes increases with their electron-accepting properties. The reactions of ArNO with GSH proceed with the formation of semimercaptal (ArN(OH)-SG) intermediate, which may rearrange into sulfinamide (ArNH-(O)SG), whose acid hydrolysis will yield ArNH_2_ or further oxidize GSH [43].

ArNHOH are relatively unstable in aqueous medium. Under aerobic conditions, the products of decomposition of phenylhydroxylamine are nitrosobenzene, nitrobenzene, and nitrophenol [44]. ArNHOH can also disproportionate yielding ArNO and ArNH_2_ [45].

**Scheme 1:**
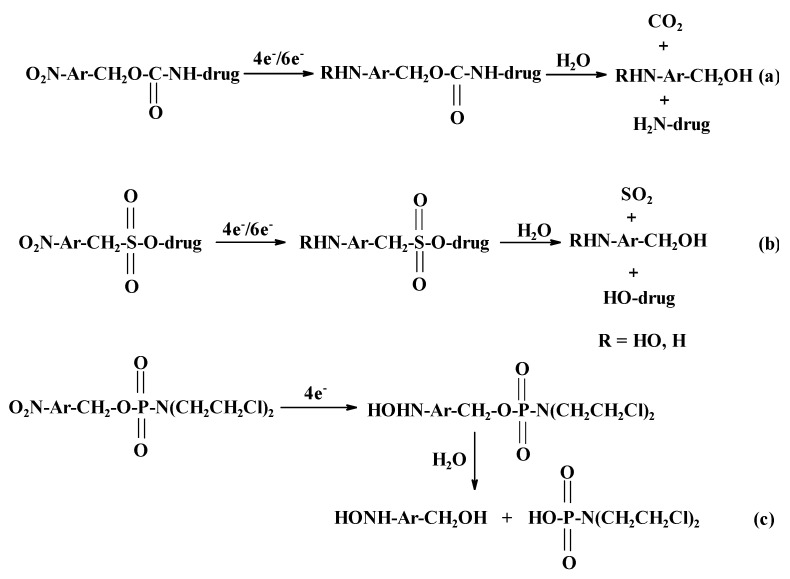
Fragmentation of reduction products of nitrobenzyl carbamates (**a**), nitrobenzyl sulfonates (**b**), and nitrobenzyl phosphoramide mustards (**c**). Adapted from the work of [47,48,49,50,51].

The mechanisms of alkylation of DNA by ArNHOH, especially by the reduction products of bifunctional dinitrobenzenes CB-1954 (**14**) and SN-23682 (**19**) and their homologues, were thoroughly analyzed and reported elsewhere [7,46], therefore, will not be addressed in this review. Another type of reaction is the fragmentation of ArNHOH with leaving groups containing a drug or chromophore molecule (Scheme 1). Because the –NHOH or –NH_2_ groups possess electron-donating properties, the reduction products hydrolyze or react with nucleophiles more easily than the parent nitroaromatics. This approach is used in the design of hypoxia-selective alkylating agents, gene-directed therapy involving nitroreductases, hypoxic tumour imaging, or imaging of nitroreductases in transfected tumors [47,48,49,50,51].

The energetics of formation of ArNH_2_ from ArNHOH in aqueous medium is also incompletely characterized. At metal or carbonaceous electrodes, nitrobenzene is reduced into phenylhydroxylamine with half-wave potentials (*E*_1/2_) of −0.30–−0.45 V, whereas the latter is reduced into aniline with *E*_1/2_ = −0.55–−0.70 V at pH 7.0 [52]. However, the overvoltage of this reaction depends on the electrode material [53]. On the other hand, the second electron transfer in this reaction, reduction in aniline radical into aniline, is characterized by *E*^1^ = 1.03 V at pH 6.9 [10]. Thus, in spite of the uncertain value of *E*^0^_7_ of phenylhydroxylamine/aniline redox couple, it is clear that the reduction in phenylhydroxylamine into aniline radical should proceed at very negative potential. This may impose certain barriers toward the enzymatic formation of ArNH_2_ from ArNHOH, in particular, single-electron transfer steps.

## 3. Mechanisms of Reduction in Nitroaromatic Compounds by Flavoenzymes

An early study of nonenzymatic reduction in nitroaromatics by reduced FMN under anaerobic conditions demonstrated a linear dependence of log *k* on *E*^1^_7_ of ArNO_2_ [54]. Its extrapolation to ∆*E*^1^_7_ = 0 gives *k* ~ 10^7^ M^−1^s^−1^, which agrees with an “outer-sphere” electron transfer model (Appendix B). The products of the reduction in nitroaromatics were hydroxylamines. Since that time, a substantial amount of information accumulated in this area, evidencing the diversity of reaction mechanisms, which will be analyzed in subsequent subsections.

### 3.1. Single- and Mixed Single- and Two-Electron Reduction in Nitroaromatic Compounds by Flavoenzymes Dehydrogenases-Electrontransferases

Flavoenzymes dehydrogenases-electrontransferases transform two-electron (hydride) transfer into a single-electron one, and, most frequently, possess single-electron transferring redox partner, heme- or FeS-containing protein. Their action is characterized by the formation of neutral (blue) flavin semiquinone, FMNH or FADH·, as a reaction intermediate. In this section, the properties of flavohemoenzymes or heme-reducing flavoenzymes and flavoenzymes FeS reductases are discussed separately. This is related not to the different properties or action mechanisms of their flavin cofactors but to the different roles of the heme or FeS redox centers in the reduction in nitroaromatics.

NADPH: cytochrome P-450 reductase (P-450R) is a 78 kD enzyme associated with the endoplasmic reticulum of a variety of eukaryotic cells. It is responsible for electron transfer from NADPH to the cytochromes P-450 and to other microsomal enzyme systems ([55], and references therein). Rat liver P-450R has a hydrophobic 6 kD *N*-terminal membrane-binding domain, the FMN-binding domain next to it, the connecting domain, and the FAD- and NADPH-binding domains at the *C*-terminal side [56]. In catalysis, the transfer of redox equivalents follows the pathway NADPH → FAD → FMN → cytochrome P-450 (cytochrome *c*). The pyrimidine part of the isoalloxazine ring of FMN is accessible to solvent. The edge-to-edge distance between the isoalloxazine rings of FMN and FAD is 3.5–4.5 Å. The negatively charged FMN-binding domain and hydrophobic membrane-binding domain are involved in the complex formation between P-450R and cytochromes P-450 or cytochrome *c*. The potentiometric and kinetic characteristics of P-450R are presented in Table 1. The large differences between the redox potential values for the first and second electron transfer point to the high stability of flavin semiquinones. A catalytic cycle involving one-, two-, and three-electron-reduced states of reductase with FMNH_2_ acting as the principal donor of electrons to oxidants is proposed [57]. It is suggested that the rates of interflavin electron transfer are higher or similar to the rate of FAD reduction by NADPH [58]. Cytochromes P-450 reoxidize P-450R with low rates, 0.15–1.0 s^−1^ [59].

P-450R reduces nitroaromatic compounds in a single-electron way and is the enzyme used most frequently to demonstrate their redox cycling reactions. For this reaction, the linear dependence of logarithms of reaction rate or *k*_cat_/*K*_m_ on *E*^1^_7_ of ArNO_2_ was observed [68,69]. This is in line with an “outer-sphere” electron transfer model [28]. Moreover, ArNO_2_ are systematically less reactive than quinones with the same *E*^1^_7_ values [69,70,71]. This additionally supports an “outer-sphere” reaction model because the electron self-exchange constants for ArNO_2_ are by two orders of magnitude lower than those for quinones [25,26,29]. Subsequently, the linear log (*k*_cat_/*K*_m_) vs. *E*^1^_7_ relationships were used for the estimation of unknown *E*^1^_7_ values of nitroaromatics [11,12,13].

The redox partners of P-450R, cytochromes P-450, catalyze the denitration of ArNO_2_ with the formation of corresponding hydroxyl derivatives [72,73] and reverse the accumulation of the amine products of polinitrobenzene reduction, catalyzing the formation of their hydroxylamines [74]. On the other hand, cytochrome P-450 101A1 is able to reduce *m*-nitroacetophenone into a corresponding amine [75]. However, the impact of these reactions on the cytotoxicity of ArNO_2_ has not been studied in detail.

Nitric oxide synthases (NOS) are dimeric flavohemoproteins that catalyze the conversion of *L*-arginine to citrulline and nitric oxide (NO·) at the expense of NADPH. Each monomer of NOS consists of a heme domain with tetrahydrobiopterin bound at its *N*-terminus and a FAD- and FMN-containing reductase domain at its *C*-terminus. The reductase and by domains are linked by a calmodulin (CAM)-binding sequence ([76], and references therein). The reductase domain is highly similar to P-450R. In catalysis, the redox equivalents are transferred in the pathway NADPH → FAD → FMN → heme (tetrahydrobiopterin), involving mainly one- and three-electron reduced states of the reductase domain in the turnover ([77,78], and references therein). The potentiometric and kinetic characteristics of neuronal NOS are presented in Table 1. Although these data may vary in various publications, it is accepted that the redox potential of cofactors decreases in the order FMN/FMNH·>> FMNH·/FMNH_2_ ≥ FAD/FADH > FADH·/FADH_2_ ≥ heme, and that the flavins are reduced much faster than heme. NOS reduces quinones and ArNO_2_ in a single-electron way via FMNH_2_ [79,80,81]. The reasons why the heme moiety is not involved in the reduction process are unclear. It may be partly explained by an increase in its redox potential after the binding of ligands. Under anaerobic conditions, nilutamide (**15**) and CB-1954 (**14**) are reduced into corresponding hydroxylamines [80,81]. TNT (**4**) and dinitrobenzenes inhibit the formation of NO· by NOS [82,83], most possibly by trapping of NO· with the product of their redox cycling, O_2_^−^, resulting in the formation of peroxynitrite. An alternative mechanism is the diversion of electron flux from FMNH_2_ to heme. Like in P-450R-catalyzed reactions, the reactivity of ArNO_2_ was characterized by the linear log *k*_cat_/*K*_m_ vs. *E*^1^_7_ relationship with some possible discrimination against the negatively charged compounds [64]. ArNO_2_ were less reactive than quinones with the same *E*^1^_7_ values.

Flavohemoglobins (FHbs) have been found in a wide variety of bacteria and fungi and play a key role in their resistance to nitrosative stress. They consist of an *N*-terminal heme-binding domain and of *C*-terminal FAD- and NAD(H)-binding modules. During turnover, NADH reduces FAD, which further reduces the Fe^3+^ form of hemoglobin (HbFe^3+^); oxyhemoglobin (HbFe^2+^O_2_) is finally formed under aerobic conditions. The reaction of the HbFe^2+^O_2_ with NO· leads to NO· detoxification, i.e., the formation of nitrate instead of the toxic peroxynitrite (ONOO^−^). The reactions proceed with a high turnover rate, ca. 100 s^−1^ [84]. The crystal structures of FHb from various sources show that the pyrimidine ring of the FAD isoalloxazine is partly accessible to solvent, whereas the access to heme may be partly hampered by a bound phospholipid molecule **66** [85]. The potentiometric and kinetic characteristics of FHb are given in Table 1. The steady-state reduction in quinones or ArNO_2_ by *S. aureus* FHb follows a “ping-pong” mechanism with the oxidative half-reaction as a rate-limiting catalysis step [67]. During the turnover in the presence of an oxidant, the reduced FAD is reoxidized by >10 times more rapidly than HbFe^2+^O_2_ moiety, i.e., it acts as a preferred electron donor. The reoxidation of heme may be hampered by a bound phospholipid molecule; moreover, the binding of O_2_ may significantly increase the potential of Fe^3+^/Fe^2+^O_2_ couple. The log *k*_cat_/*K*_m_ of nitrobenzenes and nitrofurans displays a well-expressed parabolic dependence on their *E*^1^_7_. In contrast to reactions of other electrontransferases, TNT and *p*-nitrobenzaldehyde were reduced with a 35%–40% single-electron flux. The mixed character of reduction is possibly determined by the relatively low stability of the FAD semiquinone state of FHb, 15% at equilibrium [65].

**Scheme 2:**
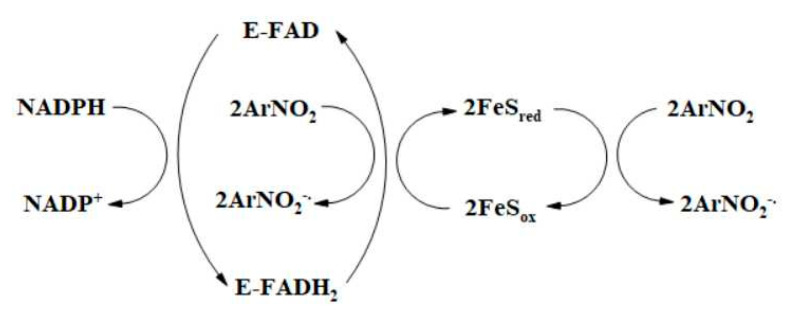
A general scheme of reduction in nitroaromatics by FeS reductases and their FeS redox partners.

Flavoenzymes FeS reductases either have the FeS proteins as redox partners or perform the intramolecular electron transfer to a FeS redox center. Some of their representatives may transfer electrons in both directions to and from FeS centers. Typically, both FeS reductases and their redox partners participate in the reduction in ArNO_2_ and other groups of redox-active xenobiotics (Scheme 2):

NADPH:adrenodoxin reductase (ADR) is a monomeric 51 kD FAD-containing enzyme, first isolated from bovine adrenal cortex mitochondria. It reduces 13.3 kD Fe_2_S_2_ protein adrenodoxin (ADX), which in turn reduces mitochondrial cytochromes P-450 that participate in the biosynthesis of steroid hormones. The ADR-ADX complex formation is determined by the electrostatic interaction between the positively charged amino acid residues of ADR and negatively charged residues of ADX ([86], and references therein). The potentiometric and kinetic characteristics of ADR and ADX are presented in Table 2.

NADPH reduces ADR with the final formation of the FADH_2_-NADP^+^ charge-transfer complex (*K*_d_~10^−8^ M). NADP^+^ also binds to the oxidized and semiquinone form of ADR but with a lower affinity. NADPH binds to the FAD semiquinone more efficiently than NADP^+^ [99].

The steady-state reactions of quinone or ArNO_2_ reduction by ADR proceed with *k*_cat_ = 20–25 s^−1^, which is close to the maximal rate of enzyme reduction by NADPH [100]. However, NADPH acts as a strong (*K*_i_ = 5.0–6.0 µM) inhibitor with respect to oxidants. Calculated by extrapolation to [NADPH] = 0, *k*_cat_/*K*_m_ values for the reduction in nitrofurans by ADR are low, 1.0 ÷ 4.0 × 10^3^ M^−1^s^−1^ [101]. It was concluded that quinones and ArNO_2_ oxidize free enzyme form and its complexes with NADPH and NADP^+^, although with slower rates. Most probably, the rate-limiting step of this reaction is the oxidation of FAD semiquinone. On the other hand, ADX stimulates the reduction in ArNO_2_ by ADR, eliminating the inhibition by NADPH and providing a more efficient alternative reduction pathway via reduced ADX [100,101]. The *k*_cat_/*K*_m_ values of nitroaromatics are much higher than in the oxidation of ADR. Their logarithms increase with their *E*^1^_7_; moreover, ArNO_2_ are less reactive than quinones with the same *E*^1^_7_ values [70,71].

*Mycobacterium tuberculosis* contains ADX-type 50 kD NADPH-feredoxin reductase FprA [102]. The *E*^0^_7_ of FAD is equal to −0.235 V [103]. The rate of FAD reduction by NADPH exceeds 160 s^−1^; however, the reaction analysis is complicated by the enzyme reoxidation by NADP^+^. Blue FAD semiquinone is formed during the reduction in the enzyme by NADPH or during its photoreduction in the presence of NADP^+^. The enzyme reduces *M. smegmatis* Fe_7_S_7_ ferredoxin with *k*_cat_ = 3.4 s^−1^ [102]. However, the data on the reactions of FprA and its redox partner(s) with ArNO_2_ are absent.

Most ferredoxin:NADP(H) oxidoreductases (FNRs) are 35–36 kD monomeric FAD-containing enzymes that transfer electrons from reduced FeS-protein ferredoxin (Fd) to NADP^+^, thus providing NADPH for CO_2_ assimilation in plants and cyanobacteria. However, some FNRs act in the opposite direction, reducing Fd at the expense of NADPH, thus supplying reduced Fd for nitrate assimilation (roots) or biosynthesis of isoprenoids (malaria parasite *Plasmodium falciparum*).

**Scheme 3:**
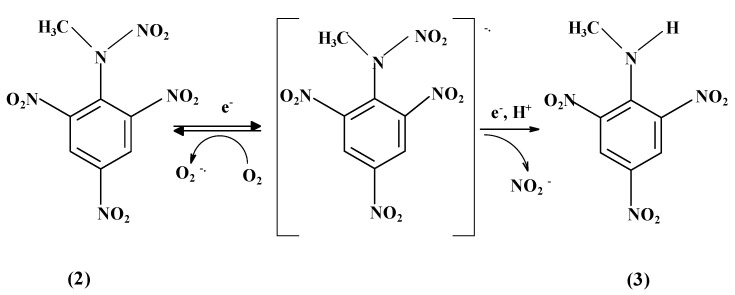
Reductive denitration of tetryl (**2**) into *N*-methylpicramide (**3**).

In this part, we discuss the mechanisms of ArNO_2_ reduction by enzymes that function in opposite directions, FNR from cyanobacterium *Anabaena* PCC7119 and from *P. falciparum* (*Pf*FNR). Their potentiometric and kinetic characteristics are presented in Table 2. The isoalloxazine ring of FAD of both FNRs is partly exposed to solvent [104,105,106]. Electrostatic interaction between the negatively charged Fd and positively charged FNR and hydrophobic interactions are involved in the formation of the *Anabaena* FNR-Fd complex [89]. An analogous electrostatic complex is formed between *Pf*FNR and *Pf*Fd [105]. Both enzymes reduce quinones and ArNO_2_ in a single-electron way [107,108,109]. Reactions follow the “ping-pong” mechanism with the oxidative half-reaction as a partly rate-limiting step, 100 s^−1^ for *Anabaena* FNR, and up to 63 s^−1^ for *Pf*FNR. They are characterized by parabolic dependences of log *k*_cat_/*K*_m_ of quinones and nitroaromatics on their *E*^1^_7_ value. Again, ArNO_2_ comprised a separate series of compounds with lower reactivity. In both enzymes, the rate-limiting step in FADH^−^ reoxidation is the oxidation of FADH·. Like in reactions of ADR, Fd stimulated the reduction in nonphysiological oxidants by both FNRs, providing an alternative reduction pathway with lower *k*_cat_ and higher *k*_cat_/*K*_m_ of oxidants. In another study, several low-potential nitroimidazoles were found to oxidize reduced *Anabaena* Fd with *k* = 630 ÷ 3500 M^−1^s^−1^ [110], which was much faster than the reduction in their analogs by FNR alone [102]. *Pf*Fd also stimulated the reduction in quinones and nitroaromatics by *Pf*FNR, providing the faster pathway of their reduction with *k*_cat_ equal to the rate of *Pf*FNR-*Pf*Fd electron transfer. Like spinach FNR, both *Anabaena* FNR and *Pf*FNR catalyze the reductive denitration of tetryl (**2**) into *N*-methylpicramide (**3**) accompanied by redox cycling of free radical intermediate [111] (Scheme 3):

The above data indicate that although *Pf*FNR possesses low homology, 20%–30%, with plant FNRs and functions in the opposite direction, its nitroreductase reaction mechanism and absence of substrate specificity are the same as of *Anabaena* FNR. However, because Fds possess higher nitroreduction rates, an important but insufficiently studied problem is their possible nitroaromatic substrate specificity.

NADH:ubiquinone reductase (CoQR, complex I) is localized in the inner mitochondrial membrane. It is a large (1 MD) enzyme of 45 subunits, catalyzing NADH oxidation by ubiquinone and performing transmembrane proton translocation. The data of numerous studies of bovine mitochondrial complex I [94,98,112,113,114] may be summarized as follows: (i) FMN and 8 FeS clusters N1-6, separated by 7.6–14 Å distances, are localized in the hydrophilic arm of L-shaped complex I that extends into the mitochondrial matrix. FMN is located in 51 kD subunit; (ii) the hydrophobic domain of the complex, localized in the inner mitochondrial membrane, pumps 4 protons from the matrix to intermembrane mitochondrial space per molecule of NADH oxidized; and (iii) the sequence of transfer of redox equivalents is NADH → FMN → N3(N1a) → N1b → N4 → N5 → N6a → N6b → N2 → bound ubiquinone. The reduction in ubiquinone is inhibited by tightly binding rotenone and piericidin. The potentiometric and kinetic characteristics of bovine complex I are presented in Table 3.

The mechanism of reduction in soluble quinones, nitroaromatics, and other artificial electron acceptors by CoQR is still a matter of debate. In this context, the reference reaction is its reduction in ferricyanide, where ferricyanide presumably directly oxidizes reduced FMN [96,97]. This reaction proceeds according to a “ping-pong” mechanism with double competitive substrate inhibition, which shows that both substrates compete for the same binding site in reduced and oxidized enzyme form. The use of 4-*S*−^2^H- NADH decreases *k*_cat_ of reaction and *k*_cat_/*K*_m_ of NADH by two times, which shows that the rate-limiting step of the process is the reduction in FMN by NADH. The reduction in soluble quinones and nitroaromatics by complex I is insensitive to rotenone and is characterized by a common parabolic dependence of log *k*_cat_/*K*_m_ on *E*^1^_7_ of oxidants. For the most active oxidants, *k*_cat_ of reaction reaches 100 s^−1^. Importantly, ArNO_2_ are reduced in a mixed single- and two-electron way with a single-electron flux of 45%–70%. The studies of the complex I inhibition by NADH, NAD^+^, redox inactive ADP-ribose, and slowly reacting quinones enabled us to conclude that quinones and nitroaromatics bind close to the ferricyanide binding site [115,116,117]. In contrast to ferricyanide, they may oxidize both free enzyme form and its complexes with NADH (*K*_d_ = 3.0 µM) and NAD^+^ (*K*_d_ = 30–60 µM), although with slower rates. Because the above *K*_d_ differ from NADH and NAD^+^ inhibition constants toward ferricyanide, it is possible that ferricyanide and quinones or ArNO_2_ oxidize different redox states of the enzyme. The possible involvement of FeS centers in nitroreduction warrants further studies.

Among the similar redox systems that may contribute to cytotoxic/therapeutic action of ArNO_2_, *Trichomonas vaginalis* contain a partly characterized Fd-dependent system. *T. vaginalis* ferredoxin (*E*^1^_7_ = −0.347 V) plays a central role in hydrogenosomal electron transport, reversibly transferring electrons from pyruvate:ferredoxin oxidoreductase (PFOR) to hydrogenase or to the NADH dehydrogenase module that contains FMN in 51 kD subunit, and Fe_2_S_2_ cluster in 24 kD subunit (FOR) [118,119,120]. Hypothetically, FOR can reduce nitroaromatics; however, the data on its nitroreductase reactions are absent. On the other hand, using the hydrogenosomal extracts of *T. vaginalis*, PFOR catalyzed pyruvate-dependent reduction in a series of ArNO_2_ (*E*^1^_7_ = −0.564 V–−0.243 V) under anaerobic conditions [121]. At fixed compound concentration, a linear log (reduction rate) vs. *E*^1^_7_ relationship is observed. *T. vaginalis* Fd stimulated the reduction in ArNO_2_; however, the reaction rate almost did not depend on *E*^1^_7_. Furthermore, it has been shown that *T. vaginalis* Fd reduces low-potential metronidazole (**40**) and other nitroimidazoles with an unexpectedly high rate, *k* = 4.2 × 10^5^ ÷ 1.0 × 10^6^ M^−1^s^−1^ [110]. On the other hand, metronidazole and another low-potential compound, chloramphenicol (**23**), are also rapidly reduced by another NADH oxidizing 26 kD FMN and FeS-containing protein, with *k*_cat_ = 56 s^−1^ and *k*_cat_/*K*_m_ = 2.0 × 10^6^ M^−1^s^−1^, and *k*_cat_ = 130 s^−1^ and *k*_cat_/*K*_m_ = 1.7 × 10^6^ M^−1^s^−1^, respectively [122]. The functions of this protein are unknown.

Microaerophilic bacterium *Helicobacter pylori* contains a similar partly characterized system, consisting of PFOR and flavodoxin:quinone oxidoreductase (FqrB) [123]. The electrons between these flavoenzymes are reversibly transferred by a low-potential electron carrier flavoprotein flavodoxin. Importantly, the reduction in NADP^+^ by FqrB was inhibited by nitrothiazole nitazoxanide (**52**) and a number of nitrochromanes, nitroben- zenes, and nitrobenzoxadiazoles, which were binding to flavodoxin [124]. The system consisting of PFOR, ferredoxin:NAD^+^ reductase, and ferredoxin, the latter participating in ArNO_2_ reduction, is also present in *Giardia* spp. [125]. However, the catalytic properties of its components are insufficiently characterized.

Mammalian xanthine oxidase (XOD) attracted some attention as a model system for the single-electron reduction in ArNO_2_. The reactions with nitroimidazoles [126] and nitroacridines [127] were characterized by the absence of structure specificity, i.e., an increase in log (reaction rate) with *E*^1^_7_ of oxidants. However, one may note that XOD is a product of proteolysis of native NAD^+^-reducing xanthine dehydrogenase (XDH) under a variety of pathophysiological conditions ([128], and references therein). While XDH prevails intracellularly, XOD is prevalent in body fluids such as milk and plasma, where it may be secreted or released from dead cells. XDH is a 2 × 145 kD dimer, with each subunit containing molybdopterin cofactor, FAD, and two Fe_2_S_2_ clusters. During the catalysis, electrons are transferred from the purine substrate to molybdopterin, then to FAD via FeS clusters, and ultimately to the final electron acceptor, NAD^+^ (XDH) or O_2_ (XOD). The rate-limiting catalysis step is the reductive half-reaction [129]. Partly purified XDH under aerobic conditions reduces nitrofurazone into several products, including its amino metabolite [130]. The fractions of XDH and XOD in the cytosol under anaerobiosis reduced 1- and 2-nitropyrenes and 4-nitrobiphenyl into their amino metabolites [131]. However, the studies of nitroreductase reactions of XDH did not receive further attention.

Summing up, the single-electron reduction in ArNO_2_ by P-450, NOS, and FNR may be attributed to the high stability of their flavin semiquinone state. Evidently, the reaction follows an “outer-sphere” electron transfer mechanism. The distances of electron transfer (*R*_p_) calculated according to this model (Appendix B, Equation (A3)), are equal to 4.2 (P-450R), 3.9 (nNOS), 4.4 (*Anabaena* FNR), 4.9–5.6 (*Pf*FNR) [109], and 2.1–3.7 Å (bovine ADX) [71,101]. These orientational values are consistent with the partial exposure of their redox centers to solvent. In all these cases, however, the principal factor determining the reactivity of ArNO_2_ is their *E*^1^_7_. This leaves relatively little space for the improvement of the enzymatic reactivity of compounds. The reasons for the mixed single- and two-electron way of ArNO_2_ reduction by CoQR and FHb are unclear. Because of the limited amount of data, the factors determining nitroaromatic oxidant specificity for the single-electron transferring flavoenzymes of *M. tuberculosis, T. vaginalis, H. pylori,* and *Giardia* spp. are unclear. On the other hand, the application of Equation (A3) in the analysis of reactions of *T. vaginalis* Fd [110] gives *R*_p_ ≤ 1.5 Å, which points to strong electronic coupling and deviation from an “outer-sphere” electron transfer model. This is in accordance with the possible binding of ArNO_2_ at the unique cavity near the FeS cluster of *T. vaginalis* Fd ([110], and references therein) and points to the possible substrate structure specificity.

### 3.2. Two-Electron Reduction in Nitroaromatic Compounds by NAD(P)H:Quinone Oxidoreductase (NQO1) and Bacterial Nitroreductases

Mammalian NAD(P)H:quinone oxidoreductase (NQO1, DT-diaphorase) is a dimeric 2 × 30 kD enzyme containing one molecule of FAD per subunit. It catalyzes two-electron reduction in quinones and ArNO_2_ at the expense of NADH or NADPH. The physiological functions of NQO1 are incompletely understood. It is supposed that it maintains vitamins K1, K2, and K3 in a reduced state and participates in the stabilization of transcription factor p53 ([132], and references therein). The activity of NQO1 is frequently elevated in various tumors ([133], and references therein). For this reason, there exists a continuing interest in the development of NQO1-directed bioreductive drugs. The studies of rat, mice, and human NQO1 revealed that the bound nicotinamide ring of NADP(H) displaces Phe178′, interacts with Tyr126′ and Tyr128′, and is stacked with FAD isoalloxazine ring at a distance of 3.4 Å. Duroquinone, other quinones, and dicoumarol, competitive to NAD(P)H, occupy the nicotinamide binding site [134,135,136,137]. In contrast, another inhibitor, cibacron blue, binds at the adenosine binding site and almost does not interact with the nicotinamide binding site. The potentiometric and kinetic characteristics of NQO1 are given in Table 3. Under redox equilibrium, NQO1 contains 8% red (anionic) semiquinone. During the reoxidation of reduced NQO1 by quinones and single-electron acceptor ferricyanide, the transient semiquinone formation was not observed. It means that the rate-limiting step of this process is the oxidation of two-electron reduced FAD [138].

The mechanism of reduction in quinones by NQO1 is understood better than the reduction in ArNO_2_. The reactions follow the “ping-pong” mechanism and are frequently characterized by quinone substrate inhibition due to the formation of dead-end complexes with the oxidized enzyme. The reactivity (log *k*_cat_/*K*_m_) of rat NQO1 with the number of quinones of different structures increased with their *E*^1^_7_ and decreased with their van der Waals volume (VdWvol) [139]. Their optimal VdWvol was estimated to be ≤200 Å^3^. The entropies of activation (ΔS^≠^) calculated according to *k*_cat_/*K*_m_ of reduction in rapidly reacting quinones were equal to −84 ÷ −76 J mol^−1^ K^−1^, whereas the reduction in “slow” quinones was characterized by ΔS^≠^ = −36 ÷ −11 J mol^−1^ K^−1^. This demonstrates more efficient electronic coupling of “fast” oxidants in the transition state [140].

**Table 3:** Potentiometric and kinetic characteristics of mammalian NAD(P)H:quinone oxidoreductase and bacterial nitroreductases.

Enzyme	Redox Potential vs. NHE, pH 7.0	Rate Constants of Electron (Hydride) Transfer, pH 7.0
Rat liver NAD(P)H:quinone oxidoreductase	−0.159 V (FAD/FADH^−^), −0.201 V (FAD/FAD^−^·) [138]	>1000 s^−1^ (NADPH to FAD) [138]; *k*_cat_ = 0.1–2300 s^−1^, *k*_cat_/*K*_m_ = 3 × 10^2^–5.4 × 10^8^ M^−1^s^−1^ (quinones, steady-state) [139]; *k*_cat_ = 0.04–75 s^−1^, *k*_cat_/*K*_m_ = 5.0 × 10^1^–5.7 × 10^5^ M^−1^s^−1^ (ArNO_2_, steady-state) [141]
*E. coli* NfsB		>1000 s^−1^ (NADH to FMN) [142]; *k*_cat_ = 225 s^−1^, *k*_cat_/*K*_m_ = 1.5 × 10^5^ M^−1^s^−1^ (nitrofurazone), *k*_cat_/*K*_m_ = 650 M^−1^s^−1^ (nitrobenzene, steady-state) [143]; *k*_cat_ = 140 s^−1^, *k*_cat_/*K*_m_ = 7.0 × 10^3^ M^−1^s^−1^ (CB-1954, steady-state) [144]; *k*_cat_ = 60 s^−1^, *k*_cat_/*K*_m_ = 1.3 × 10^4^ M^−1^s^−1^ (PR-104A, steady-state) [145]
*E. cloacae* NR-B	−0.190 V (FMN/FMNH^−^) [146]	>1000 s^−1^ (NADH to ArNO_2_, steady-state) [39]; 700 s^−1^ (NADH to FMN), 1.9 s^−1^ (FMNH^−^ to *p*-nitrobenzoic acid, 4 °C) [147]
*E. coli* NfsA	−0.215 V (FMN/FMNH^−^) [148]	>400 s^−1^ (NADPH to FMN), *k*_cat_ = 14–180 s^−1^, *k*_cat_/*K*_m_ = 10^4^–7.9 × 10^6^ M^−1^s^−1^ (ArNO_2_, steady-state) [149]
*M. smegmatis* *Ms*PnBA	−0.190 V (FMN/FMNH^−^) [150]	*k*_cat_ = 3.4–19.2 s^−1^, *k*_cat_/*K*_m_ = 1.2 × 10^3^–1.6 × 10^5^ M^−1^s^−1^ (ArNO_2_, steady-state) [150]
Peroxiredoxin- nitroreductase hybrid enzyme, *Thermotoga maritima*	−0.185 V (FMN/FMNH^−^, pH 8.0) [151]	146 s^−1^ (NADH to FMN), *k*_cat_ ≤ 15 s^−1^, *k*_cat_/*K*_m_ = 10^1^–5.8 × 10^5^ M^−1^s^−1^ (ArNO_2_, steady-state) [152]

NQO1 reduces ArNO_2_ more slowly than quinones (Table 3) [141]. For example, CB-1954 (**14**) is reduced by rat NQO1 with *k*_cat_ ≤ 0.1 s^−1^ and *k*_cat_/*K*_m_ < 10^3^ M^−1^s^−1^. The reactivity of human NQO1 is even lower because of a Tyr104Glu substitution [153]. Next, we will address the kinetic properties of rat NQO1. Its best substrates, tetryl (**2**) and tetranitrobenzimidazolone (**42**) are characterized by *k*_cat_ ≥ 70 s^−1^ and *k*_cat_/*K*_m_ > 10^5^ M^−1^s^−1^. The ΔS^≠^ of reduction in ArNO_2_ is even less negative than those of “slow” quinone substrates, which points to their less efficient electronic coupling. One should note that NQO1 performs the reductive *N*-denitration of tetryl (**2**) (Scheme 3) in a mixed single- and two-electron way [154]. This supports the possibility of a multistep (e^−^,H^+^,e^−^) hydride transfer in the reduction in other nitroaromatics with an initial single-electron transfer.

The reactivity of a series of nitrobenzimidazoles toward rat NQO1 increases with their *E*^1^_7(calc.)_ [155]. However, for a larger set of compounds, the dependence of log *k*_cat_/*K*_m_ on *E*^1^_7_ is almost absent. The reactivity of ArNO_2_ increases with the stability of their complexes with *the* oxidized form of NQO1 [141,155]. However, the criteria for efficient complex formation are poorly understood. Another factor enhancing the reactivity of ArNO_2_ is an increased torsion angle of the nitro group. This, in particular, explains why NQO1 reduces the 4-nitro group of CB-1954 (**13**). The combined studies of the effects of dicoumarol and cibacron blue and interdependent quinone and ArNO_2_ inhibition enabled us to conclude that nitroaromatics and bulky quinones bind partly outside the nicotinamide/dicoumarol binding pocket and may partly occupy adenosine/cibacron blue binding pocket [139,141].

Bacterial type I oxygen-insensitive nitroreductases (NRs) catalyze NAD(P)H-dependent multistep reduction in ArNO_2_ with the formation of nitroso, hydroxylamine and amino products. Among several groups of type I NRs, two major ones are distinguished according to their similarity with *E. coli* nitroreductases A or B [156]. The physiological functions and physiological electron acceptors for bacterial type I NRs are not clearly characterized. It is suggested that they participate in the antioxidant defense of microorganisms as part of the soxRS regulon, whose genes are upregulated in response to oxidative stress [157]. Oxygen-insensitive NRs play a considerable role in the bioreductive degradation of polynitroaromatic explosives such as 2,4,6-trinitrotoluene (TNT) (**4**) [3]. They also are potential candidates for gene-directed enzyme-produg cancer therapy (GDEPT) using nitroaromatic compounds. This approach is based on the much higher rates of two-electron reduction in ArNO_2_ by bacterial NRs than by human NQO1 [7,8,15]. In this case, genes encoding NRs are inserted into the tumor cells using either a virus or a plasmid. Further, we will address the properties of NRs with well-characterized catalysis mechanisms and/or well-established biomedical importance.

Most type I reductases are 2 × 24 ÷ 27 kD dimers, containing one FMN per subunit. They may reduce a wide spectrum of oxidants, including nitroaromatics, quinones, riboflavin derivatives, and inorganic complexes [142,158,159]. Typically, they are inhibited by micromolar or lower concentrations of dicoumarol, which binds at the dihydronicotinamide binding site of NAD(P)H, and acts as a competitive inhibitor toward NAD(P)H. Their reactions proceed according to the “ping-pong” mechanism. The potentiometric and kinetic characteristics of NRs are given in Table 3.

The best-characterized members of group B NRs are *E. coli* nitroreductase B (NfsB) and *Enterobacter cloacae* nitroreductase (*E. cloacae* NR). The X-ray studies of NfsB show that the FMN isoalloxazine ring is localized in the intersubunit domain, its *re*-plane is solvent-accessible [143,160,161]. The nicotinamide ring of bound NAD(P) is stacked between isoalloxazine and Phe124‘. Nitroaromatic compounds bind close to the nicotinamide binding domain of NfsB; however, there exist several potential binding sites of CB-1954 (**14**), with the participation of Lys14, Lys74, Ser12, or Phe124, Asn71, and Gly166 [150]. On the other hand, nitrofurazone interacts with Glu165 and Phe70 and binds in nonproductive conformation because its nitrofuran group does not stack over the isoalloxazine ring of FMN [143]. This may point to the flexibility of the active center of NfsB during catalysis and to its ability to accommodate the oxidants of different sizes and shapes. This is particularly indicated by the ability of NfsB to reduce either the 2- or 4-nitro group of CB-1954 and only the 2-nitro group of SN-23682 (**19**) [162]. The kinetic parameters of NfsB (Table 3) show that it reduces CB-1954 much more rapidly than does NQO1. As the maximal rates of reduction in nitroaromatics are lower than the rate of reduction in FMN (Table 3), the oxidative half-reaction is the rate-limiting step of catalysis of NfsB. The early studies [158,159] point to the possible absence of substrate specificity and the increase in their reactivity with reduction potential. According to the studies of a series of derivatives of CB-1954 and SN-23682, their *k*_cat_/*K*_m_ varied in the range of 4.8 × 10^2^–6.3 × 10^4^ M^−1^s^−1^ [162]. This data scattering points to a definite effect of the size and position of substituents on the reactivity of compounds, which, however, is difficult to characterize. On the other hand, the reactivity of compounds starts to decrease at their VdWvol ≥ 400 Å^3^.

*E. cloacae* NR possesses 88% homology with NfsB [163]. The potentiometric and kinetic characteristics of the enzyme are given in Table 4. The semiquinone state of FMN of *E. cloacae* NR is extremely unstable, ca. 0.01% at equilibrium [146]. This possibly determines the two-electron character of reduction in ArNO_2_. The oxidative half-reaction is the rate-limiting step of the catalytical cycle. Using 4-*R*–^2^H-NADH, kinetic isotope effects are observed in both reductive and oxidative half-reactions [147]. It shows that the H atom, transferred from dihydronicotinamide to N-5 position of isoalloxazine, is subsequently transferred to oxidant during two-electron reduction if the exchange of proton at N-5 position with the solvent is sufficiently slow. In contrast with single-electron transferring flavoenzymes and NQO1, *E. cloacae* NR reduced ArNO_2_ faster than quinones with the same *E*^1^_7_ value [39,164]. The reactivity of nitroaromatics increased with their *E*^1^_7_ or correlated with their ∆Hf(ArN(OH)O^−^) or ∆Hf(ArNO_2_^−^), thus showing little structure specificity. The *k*_cat_/*K*_m_ of the most efficient oxidants, derivatives of tetryl (**2**), reached 10^7^ M^−1^s^−1^. TNT (**4**) oxidized 4 NADH equivalents in two steps, apparently being reduced to dihydroxylamino derivative, whereas tetryl oxidized 6 NADH equivalents. Importantly, *E. cloacae* NR did not catalyze reductive *N*-denitration of tetryl (Scheme 3) and reduced it into currently unidentified products.

*Salmonella typhimurium* NR is a 2 × 24 kD dimer containing FMN and possessing 89% identity with *E. cloacae* NR [165]. It reduces nitrobenzene derivatives with *k*_cat_ = 5.8–290 s^−1^ and *k*_cat_/*K*_m_ = 1.7 × 10^3^–3.7 × 10^5^ M^−1^s^−1^ [150]. The reactivity of nitrobenzenes increases with the σ value of their substituents.

**Scheme 4:**
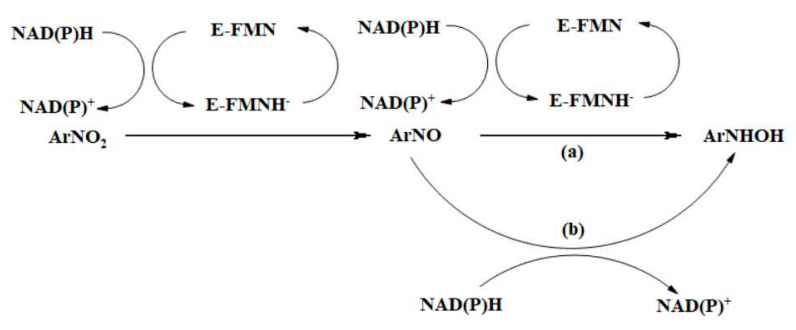
A general scheme of reduction in nitroaromatics by oxygen-insensitive nitroreductases involving an enzymatic (pathway (**a**)) and nonenzymatic (pathway (**b**)) reduction in nitroso intermediate.

**Figure 4:**
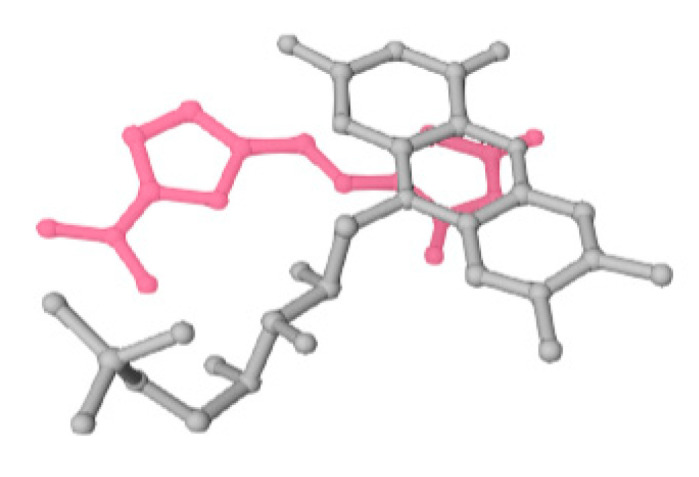
The orientation of nitrofurantoin (**pink**) and FMN (**gray**) in the complex of nitrofurantoin with oxidized *E. coli* NfsA. The structure is taken from the Protein Data Bank, accession code 7NB9 [142].

*E. coli* NfsA, an FMN-dependent NADPH-specific enzyme, is the best-characterized member of group A nitroreductases [166]. The kinetic and potentiometric characteristics of the enzyme are given in Table 3. NfsA follows the “ping-pong” mechanism with the rate-limiting oxidative half-reaction [149]. The reactivity of ArNO_2_ is systematically higher than the reactivity of quinones possessing the same *E*^1^_7_. NfsA reduced tetryl (**2**) to the same unidentified products as did *E. cloacae* NR. The second step of a net four-electron reduction in ArNO_2_, the formation of ArNHOH from ArNO intermediate, is most likely the direct nonenzymatic reduction in ArNO by NADPH (Scheme 4, pathway (b)) [41]:

Like in NfsA, nitrofurantoin binds at the active center of NfsA in nonproductive orientation (Figure 4), its nitrofuran ring interacts with Arg15 and Lys167, and its imidazole group binding over the isoalloxazine ring interacts with Arg225 [142]. This shows that the catalysis of NfsA, NfsB, and *E. cloacae* NR may share some common features. However, there may exist certain differences in their action. The computer modeling study suggests that the binding CB-1954 to NfsA involves Ser40 (Ser41 in NfsB) and Ile129′ (Phe124′ in NfsB); however, it may also involve Phe42 that is absent in NfsB [167]. In contrast to *E. cloacae* NR and NfsB, dicoumarol does not quench the fluorescence of FMN, which points to its relatively weak interaction with isoalloxazine [149].

In NfsA-catalyzed reactions, log *k*_cat_/*K*_m_ of a series of nitrobenzenes including CB-1954 and nitrofurans correlated well with their *E*^1^_7_ [149]. The *k*_cat_/*K*_m_ of the most efficient oxidant, tetryl, reaches 7.9 × 10^6^ M^−1^s^−1^. The reduction rate constants of several 2-nitroimidazoles (*E*^1^_7_ ~ −0.390 V), dinitrobenzene PR-104 (**13**), and metronidazole (**40**) obtained in other studies [15,168,169] are also close to this correlation. Thus, the data available so far demonstrate that the reactivity is determined mainly by the reduction potential of ArNO_2_ and not by their structural peculiarities.

*Mycobacterium smegmatis* enzyme *Ms*PnBA is classified as group A nitroreductase [150]. The kinetic and potentiometric characteristics of the enzyme are presented in Table 3. This nitroreductase reduces antitubercular benzothiazinones into their amines and confers *M. smegmatis* resistance to these drugs [170]. Like in NfsA-catalyzed reactions, the reactivity of a series of examined nitrobenzenes increases with the σ value of their substituents [150]. Other less-characterized NfsA-like nitroreductases from *Neisseria meningitidis* and *Bartonella henselae* reduce CB-1954 and metronidazole with similar rates to those of NfsA [169].

A relatively well-characterized oxygen-insensitive NR is the peroxiredoxin- nitroreductase (Prx-NR) hybrid enzyme of *Thermotoga maritima*, which consists of a Prx domain at the N-terminus, and an FMN-containing NR domain at the C-terminus. These domains function independently without the exchange of redox equivalents [151]. The NR domain of Prx-NR (residues 142–321) possesses a 20–24% homology with *E. coli* NfsB and *E. cloacae* NR, and 18% homology with NfsA [152], and does not contain the residues analogous to Phe124′, Phe70, Ser40, Lys14 and Lys74 of group B NRs. The rate-limiting step of Prx-NR catalysis is the oxidative half-reaction. Importantly, the substrate specificity of Prx-NR differs from that of *E. cloacae* NR and NfsA. Although the log *k*_cat_/*K*_m_ of ArNO_2_ increased with their *E*^1^_7_, nitroaromatics were less reactive than quinones with the same *E*^1^_7_ values [152]. Because this phenomenon is characteristic of single-electron reduction (see Section 3.1), it is possible that ArNO_2_ are reduced in a multistep (e^−^, H^+^, e^−^) way with a rate-limiting first electron transfer.

*Helicobacter pylori* contains nitroreductase RdxA, an FMN-containing 2 × 26 kD dimer that shows no more than 29% sequence identity with other homologous structures of NRs [171,172]. RdxA exhibits high NADPH oxidase activity, 2.8 s^−1^. The maximal reduction rate of nitrofurazone and CB-1954 was similar to RdxA oxidase activity. They were reduced with *k*_at_/*K*_m_ of 1.4 × 10^6^ M^−1^s^−1^ and 3.0 × 10^5^ M^−1^s^−1^, respectively. Metronidazole was reduced with a much lower rate, *k*_cat_ = 0.22 ÷ 0.62 s^−1^ and *k*_at_/*K*_m_ = 2.0 × 10^3^ M^−1^s^−1^. Nitrothiazole nitazoxanide (**52**) was reduced by RdxA with a rate similar to that of metronidazole [173]. Another *H. pylori* nitroreductase, FrxA, is even more scarcely characterized. In this case, the reactivity of ArNO_2_ increases in the order metronidazole < nitrofurans < nitazoxanide [173]. The factors determining substrate specificity of both NRs are unclear.

Nitroreductase-type enzymes (NTR) were found in parasites *Trypanosoma cruzi* and *T. brucei* [174,175,176]. They represent 2 × 30 kD dimers containing one FMN per monomer. *T. cruzi* NTR is NADPH-specific, whereas the *T. brucei* enzyme oxidizes both NADH and NADPH. They are inhibited by dicoumarol with *K*_i_ = 258 nM and *K*_i_ = 14 nM, respectively. These enzymes reduce nitrofurans, nitroimidazoles, and nitrobenzenes with *k*_cat_ = 0.2 ÷ 1.2 s^−1^, and *k*_cat_/*K*_m_ ranging from 7.3 × 10^2^ to 2.5 × 10^5^ M^−1^s^−1^. The final product of reduction in nifurtimox under aerobic conditions is unsaturated open-chain nitrile, whose antitrypanosomal activity was close to that of nifurtimox. Benznidazole is metabolized into hydroxylamine products, which further undergoes secondary reactions. The factors determining enzyme-substrate specificity are unclear. It is evident that the activity of compounds does not correlate with their *E*^1^_7._

*Leishmania* spp. possess several types of NTR. An FMN-dependent mitochondrial 34.7 kD NADH and NADPH-oxidizing nitroreductase (NTR1) from *Leishmania major* reduces benznidazole, nifurtimox, CB-1954, and related compound without pronounced substrate specificity, with *k*_cat_ = 0.01 ÷ 0.07 s^−1^ and *k*_cat_/*K*_m_ = 2.5 × 10^3^ ÷ 1.0 × 10^4^ M^−1^s^−1^ [177]. This enzyme participates in the bioactivation of a novel antitrypanosomal and leishmanicidal agent fexinidazole (**39**); however, the kinetics data are not presented [178]. Because fexinidazole is a representative of 5-nitroimidazoles, one may expect its *E*^1^_7_ in the range of −0.490 ÷ 0.430 V (Table 1). Another FMN-dependent cytosolic 39.6 kD enzyme (NTR2) was identified in *L. donovani* [179]. It is specific toward bicyclic low-potential nitroimidazooxazines such as *R*-PA–824 (**57**) and its analogs, whose turnovers are equal to 2.0 ÷ 10.1 s^−1^ at 100 µM compound concentration, whereas the more powerful monocyclic oxidants nifurtimox and fexinidazole are reduced much slower, with rates of 0.12 and <0.01 s^−1^, respectively [179].

There exist limited data on the formation of amines as the final product of reduction in ArNO_2_ by type I NRs, although this problem is relevant both to biomedicine and ecotoxicology. NfrA from *Bacillus LMA* exhibits 40% homology with NfsB and reduces 3,5-dinitro-trifluoromethylbenzene to diamine product with *k*_cat_ = 18 s^−1^ [180]. Nitrofurazone is reduced to its amine derivative at a lower rate. *S. typhimurium* NR quantitatively reduces nitrobenzene into aniline [165]. It is unclear whether the tendency for amine formation is determined by the properties of ArNO_2_ or nitroreductase or by both factors. It has been suggested that the possibility of amine formation increases with the reduction potential of ArNO_2_ and the size of their aromatic system [150]. A recent study shows that *Haemophilus influenza* NR-B reduces chloroamphenicol (**23**) into a corresponding amine with *k*_cat_ = 10.2 s^−1^ and *k*_at_/*K*_m_ = 2.0 × 10^4^ M^−1^s^−1^ [181]. This NR possesses unusual and undisclosed substrate specificity because it reduces more powerful oxidant metronidazole (**40**) (Table 1) with a lower rate, *k*_cat_ = 0.34 s^−1^ and *k*_at_/*K*_m_ = 4.6 × 10^3^ M^−1^s^−1^ with the formation of its hydroxylamine metabolite.

There also exist several potentially important but insufficiently characterized flavin-independent enzymes with nitroreductase activity. In spite of the presence of nitroreductase *Ms*pnBA in *M. smegmatis* [170], this enzyme is absent in *M. tuberculosis.* In this case, the antitubercular drug *S*-PA-824 (**57**) is reduced by deazaflavin F-420 (7,8-didemethyl-8-hydroxy-5-deazariboflavin)-dependent nitroreductase [182]. This reaction with *k*_cat_ = 0.1 s^−1^ leads to the formation of NO·. Under aerobic conditions, human aldo-keto reductase 1C3 catalyzes NADPH-dependent reduction in PR-104A (**13**) into its hydroxylamino metabolite with *k*_cat_ = 0.013 s^−1^ [183].

Summing up, the two-electron reduction in ArNO_2_ by NQO1 and bacterial oxygen-insensitive NRs may be attributed to the low stability of their flavin semiquinone state. However, the relative stability of FAD^−^ of NQO1, 8% under equilibrium [138], may enable this enzyme to perform the reductive denitration of tetryl (**2**) (Scheme 3) in a mixed single- and two-electron way [143]. This reaction is not characteristic for *E. cloacae* NR-B and *E. coli* NfsA [39,149], evidently due to the much lower stability of their FMN semiquinone [146]. The crystallographic studies of NRs from *E. coli* [142,143,160,161] point to the flexibility of their active sites and to their ability to accommodate the substrates of various sizes. The kinetic studies of several A- and B-type NRs demonstrate that the reactivity of ArNO_2_ is strongly influenced by their reduction potential [39,149,150]. However, this leaves some space for the improvement of the activity of compounds. Another unresolved problem is the factors determining substrate specificity of nitroreductases from *H. pylori, H. influenza, Leishmania,* and *Trypanosoma* spp.

### 3.3. Single- and Two-Electron Reduction in Nitroaromatic Compounds by Flavoenzymes Disulfide Reductases

Flavoenzymes disulfide reductases contain FAD and redox-active disulfide group, which participate in the transfer of redox equivalents in a sequence NAD(P)H → FAD → catalytic disulfide → low-*M*_r_ or protein disulfide substrate. In most cases, they perform antioxidant functions. These reactions proceed via obligatory two-electron (hydride) transfer without the formation of free radical intermediates ([184,185], and references therein). Although being slow, the nitroreductase reactions of disulfide reductases received significant attention because of the combined action of ArNO_2_, redox cycling, and inhibition of physiological reactions of disulfide reductases. It is important to note that these compounds are reduced by flavin but not by reduced disulfide cofactor due to unfavorable energetics of single-electron oxidation of dithiols [186].

Glutathione reductase (GR) and trypanothione reductase (TR), the 2 × 55 kD homodimers, contain one FAD and catalytic disulfide per subunit. GR catalyzes the reduction in glutathione disulfide (GSSG), and TR catalyzes the reduction in trypanothione (TS_2_), a glutathione-spermidine conjugate. The structure and reaction mechanism of both enzymes are similar [187,188,189]. GR performs antioxidant functions in various organisms. TR is found exclusively in trypanosomes and leishmanias, the causative parasites of several tropical diseases, including African sleeping sickness and Chagas disease. These parasites do not contain GSSG/GSH, and their antioxidant defense relies mainly on TR-catalyzed regeneration of T(SH)_2_. The presence of amino acids with different charge in the disulfide substrate-binding domain of HGR and *T. congolense* TR [187,190] enable the discrimination between the negatively charged GSSG and positively charged TS_2_.

GR and TR are reduced by NADPH to two-electron reduced form (EH_2_), which is the FAD-thiolate charge-transfer complex with the main electron density being localized on thiolate. Next, EH_2_ is reoxidized by disulfide. These reactions follow a “ping-pong” mechanism with reductive half-reaction as a rate-limiting step. The *k*_cat_ values of human erythrocyte GR (HGR), *P. falciparum* GR (*Pf*GR), and TRs span between 120 and 240 s^−1^ [185,191,192,193,194,195]. More precisely, their mechanism should be classified as “hybrid ping-pong” because during turnover, GSSG reoxidizes not free EH_2_ form, but its tight complex with repeatedly bound NADPH (*K*_d_ = 2.1 µM, yeast GR [196]). In this case, GSSG oxidizes free EH_2_ and its complexes with NADPH and NADP^+^ with sufficiently close rates. The redox potentials of HGR, *Pf*GR and *T. congolense* TR are equal to −0.227 (pH 8.2 [193]), −0.206 (pH 6.9 [197]), and −0.275 V (pH 7.5 [198]), respectively. Under artificial conditions, GR may be further reduced into the four-electron reduced state (EH_4_); however, this form is not formed during the enzyme turnover. This is attributed to the tight binding of NADPH, which stabilizes the EH_2_ form.

ArNO_2_ are reduced by GR and TR in a single-electron way. The most efficient oxidant of HGR and *Pf*GR is tetryl (**2**) (*k*_cat_ ≥ 5 s^−1^, *k*_cat_/*K*_m_ = 2.0 ÷ 7.5 × 10^3^ M^−1^s^−1^ [195]). The low reaction rates complicate the substrate specificity studies. However, the introduction of basic substituents into nitrofuran molecule enhances their reactivity toward TR (*k*_cat_ = 2.5 ÷ 3.0 s^−1^, *k*_cat_/*K*_m_ = 3.3 ÷ 9.2 × 10^4^ M^−1^s^−1^ [199,200]). A specific feature of quinone and nitroreductase reactions of GR and TR is the activation by the reaction product NADP^+^ [192,201]. Although the main electron density in the FAD-thiolate charge-transfer complex is localized on thiolate, its minor part remains on FAD. The binding of NADP^+^ to EH_2_ with a concomitant displacement of NADPH increases the electron density on FAD, which may accelerate the reduction in xenobiotics. The order of reactivity of various redox forms of GR and TR with quinones and presumably with ArNO_2_ is EH_2_ < EH_2_-NADPH < EH_2_-NADP^+^ < EH_4_ [192,202]. However, the site(s) of their reduction are not characterized.

An important aspect of the interaction of ArNO_2_ with GR and TR is the inhibition of the reduction in their physiological disulfide oxidants [17,195,199,200,201,202,203]. In these cases, compounds act as non- or uncompetitive inhibitors with respect to NADPH and disulfide substrate and bind at the intersubunit domain of GR or TR close to the binding sites of GSSG or TS_2_. The amino acid residues of this domain of HGR, *Pf*GR, and TR are strikingly different [189,204]. Currently, significant efforts are devoted to the studies of efficient and specific inhibitors targeting this domain, with an aim to develop new antimalarial or trypanocidal drugs [194,205]. The efficiency of ArNO_2_ or other redox-active compounds is evaluated by the ratio (*k*_cat_/*K*_m_)/*K*_i_, which combines their ability to serve as redox cycling agents and inhibit GSSG/TS_2_ reduction [194]. For most active compounds, this ratio is above 10^11^ M^−2^s^−1^ (naphthoquinones) and above 10^10^ M^−2^s^−1^ (nitrofurans) [194,199,200].

Lipoamide dehydrogenase (LipDH, NADH:lipoamide oxidoreductase) is structurally similar to GR and TR, although it does not perform antioxidant functions. Mammalian LipDH catalyzes the NAD^+^-dependent oxidation of covalently bound dihydrolipoate of the pyruvate dehydrogenase or α-ketoglutarate dehydrogenase complexes [184]. Pig heart enzyme catalyzes the rapid transfer of two redox equivalents in both directions, from dihydrolipoamide to NAD^+^ and from NADH to lipoamide (LipS_2_NH_2_). In the latter case, the rate-limiting step of the process is the oxidative half-reaction [184,206]. Like GR or TR, LipDH shuttles between E_ox_ and EH_2_ states.

Pig heart and *T. cruzi* LipDH reduce nitroaromatic compounds with *k*_cat_ = 0.1 ÷ 2.0 s^−1^ [203,207] that makes 0.02%–0.5% of LipS_2_NH_2_ reduction rate. Some information on the reaction mechanism may be obtained from the studies of quinone reduction, which takes place with 20% (pig heart LipDH [206]), or even with 80% of maximal disulfide oxidant reduction rate (*M. tuberculosis* LipDH [208]). It was concluded that the EH_4_ fraction of LipDH, which is formed in the absence of NAD^+^, is mostly responsible for quinone reduction. Because *M. tuberculosis* LipDH efficiently reduces quinones, *k*_cat_ =165–190 s^−1^ [208], one may also expect its significant nitroreductase activity, which, however, was not investigated.

NAD(P)H-thioredoxin reductases (TrxRs) are important antioxidant enzymes in both prokaryotic and eukaryotic organisms [209]. There exist two groups of TrxRs: high-*M*_r_ (2 × 54–58 kD) enzymes contain FAD, catalytic disulfide, and a third cofactor, selenylsulfide (mammalians), or additional disulfide (*P. falciparum*) in their active center. Low-*M*_r_ enzymes (2 × 35 kD) from plants, bacteria, and archaea contain FAD and catalytic disulfide. The physiological substrates of TrxRs are 10–12 kD disulfide proteins thioredoxins (Trxs), and sometimes disulfide or monothiol-containing glutaredoxins (Grxs). Apart from the antioxidant action, they perform other numerous physiological functions [210,211]. It is important to note that human TrxR is overexpressed in numerous cancers [212].

First, let us discuss the properties of mammalian (rat, human) TrxR. The catalytic cycle of the enzyme is similar to GR, except for the participation of selenylsulfide, which is located at the C-end of the protein [213,214]. The rate-limiting catalysis step, 30 ÷ 40 s^−1^, is the two-electron transfer between dithiolate and selenylsulfide, which is responsible for the reduction in Trx and other numerous substrates of mammalian TrxR. During catalysis, the enzyme cycles between EH_2_ and EH_4_ redox states (*E*^0^_7_ = −0.294 V [215]). Mammalian TrxR may directly reduce 5,5^‘^-dithiobis(2-nitrobenzoic acid) (DTNB), with the maximal rate being equal to that of Trx reduction. Other less efficient oxidants are cystine, lipoate, alloxan, dehydroascorbate, vitamin E, coenzyme Q10, and vitamin K, lipid peroxides, and H_2_O_2_.

Rat TrxR reduces ArNO_2_ either in a two-electron way (*p*-dinitrobenzene) or in a single-electron way (tetryl) [216]. During the reaction, TrxR cycles between four- and two-electron reduced states. The reactivity of ArNO_2_, *k*_cat_ = ≤ 0.1–2.8 s^−1^, and *k*_cat_/*K*_m_ = < 10^2^–1.4 × 10^4^ M^−1^s^−1^ [216,217], is comparable with that of quinones, which follow poorly expressed linear log *k*_cat_/*K*_m_ vs. *E*^1^_7_ relationship [215]. The reduction in nitroaromatics that may alkylate -SH/-Se^−^ groups (1-chloro−2,4-dinitrobenzene, tetryl (**2**), dinitrobenzo- furoxane (**48**), 2-phenylsulfonylnitropyridines) is accompanied by a rapid covalent modification of the enzyme with a loss of DTNB and Trx reductase activity and to an increase in NADPH oxidase activity [216,218,219]. The most important feature of TrxR is that in addition to reduced FAD, the reduced selenylsulfide may participate in the reduction in fully substituted quinones such as phenanthrene quinone or toxoflavin [215,220], and, presumably, nitroaromatics. This was demonstrated by the selective suppression of these reactions by Au-thioglucose, which specifically reacts with selenol. The participation of selenocysteine but not cysteine in these reactions may be explained by the relative ease of its single-electron oxidation since *E*_7_(Sec·/Sec^−^) is equal to 0.43 V, whereas *E*_7_(Cys·/CysH) is equal to 0.92 V [186]. Dinitrobenzenes and nitrofurans acted as weak noncompetitive inhibitors with respect to DTNB (*K*_i_ = 17–400 µM), whereas tetryl and dinitrobenzofuroxane (**48**) acted as competitive inhibitors with *K*_i_ values of 12.5 and 5.0 µM, respectively [216]. This points to the existence of several binding sites of ArNO_2_.

Another representative of high-*M*_r_ enzymes, *P. falciparum* TrxR, contains FAD and two catalytic disulfides, Cys88, Cys93, and Cys535, Cys540 in the active center [221]. The enzyme reduces its physiological oxidant, *Pf*Trx1, and quinones with relatively high and similar rates, 51.7 and 31–67 s^−1^, respectively [222]. This is >10 times faster than the reduction in quinones by *Pf*GR or HGR. However, the nitroreductase reactions of *Pf*TrxR have not been examined.

Low-*M*_r_ TrxRs from *E.coli* and other species contain one FAD and one redox-active disulfide per subunit. In contrast with GR and TR, their FAD and disulfide redox groups function separately. The *E*^0^_7_ values of FAD/FADH_2_ and S_2_/(SH)_2_ redox couples are equal to −0.243 ÷ −0.260 and −0.254 ÷ −0.271 V, respectively [223]. In *E. coli* TrxR, NADP(H) binds at the vicinity of catalytic disulfide and at 17 Å distance from the isoalloxazine ring of FAD. In order to reduce FAD, the disulfide/NADP(H)-binding domain should undergo substantial rotation, which in turn exposes the catalytic disulfide into solution and makes it accessible to Trx [224]. This makes the formation of charge-transfer complex formation impossible [225,226]. The catalysis rate-limiting step, 25 ÷ 30 s^−1^, is either the conformational transition or Trx reduction. In catalysis, TrxR cycles between two- and four-electron reduced forms, FADH_2_/S_2_ and FADH_2_/(SH)_2_. Interestingly, in spite of an obligatory two-electron character of physiological reactions of *E. coli* TrxR, it forms neutral (blue) FAD semiquinone during irradiation under anaerobic conditions [227].

The reactions of ArNO_2_ and other prooxidant xenobiotics with *E. coli* TrxR were not studied. However, they were examined with structurally similar TrxRs from *Arabidopsis thaliana* (*E*^0^_7_(FAD/FADH_2_) = −0.244 V) [228,229,230] and *Thermotoga maritima* (*E*^0^_7_(FAD/FADH_2_) = −0.230 V) [151,231,232,233]. The latter enzyme preferentially oxidizes NADH, and its physiological oxidant is a 25.2 kD Grx-1. Although its crystal structure is unavailable, this enzyme possesses two catalytic cysteines Cys147 and Cys150, a vicinal NAD(P)H bonding motif, FAD bonding motif, and interdomain motif Gly251, Pro254, which possibly participates in domain rotation [231]. Both enzymes reduced ArNO_2_ with 70%–90% single-electron flux [230,232] and catalyzed *N*-denitration of tetryl with the formation of *N*-methylpicramide (Scheme 3). This reaction was characterized by *k*_cat_ = 1.5 s^−1^ and *k*_cat_/*K*_m_ = 2.7 × 10^4^ M^−1^s^−1^ (*A. thaliana* TrxR), and *k*_cat_ = 6.3 s^−1^ and *k*_cat_/*K*_m_ = 6.6 × 10^4^ M^−1^s^−1^ (*T. maritima* TrxR). In both cases, the log *k*_cat_/*K*_m_ of reduction in ArNO_2_ increased with *E*^1^_7_. The reactivity of nitroaromatics was by one order of magnitude lower than that of quinones with the same *E*^1^_7_ values. On the analogy with quinone reduction by *T. maritima* TrxR, the rate-limiting reaction step in nitroreductase reactions of low-*M*_r_ TrxRs may be the oxidation of FAD semiquinone [233]. It is important to note that the single-electron character of ArNO_2_ reduction and reactivity of *A.thaliana* and *T. maritima* TrxRs are similar to the properties of low-*M*_r_ TrxRs from *T. vaginalis* and *Giardia lamblia* [234,235]. Thus, they may be considered as the model enzymes for the studies of this pathway of ArNO_2_ activation in parasites.

The structure and functions of *Salmonella typhimurium* alkyl hydroperoxide reductase AhpF share some similarities both with high-*M*_r_ and low-*M*_r_ TrxRs ([236], and references therein). Its 58 kD monomer contains FAD and two redox-active disulfide centers, Cys345-Cys348 and Cys129-Cys132. *C*-terminal FAD-binding domain of AhpF possesses 34% identity with the analogous domain of *E. coli* TrxR. Through putative large interdomain movements, electrons are transferred in a sequence NADH → FAD → Cys345-Cys348 → Cys129-Cys132. The latter center, located in *N*-terminus, transfers electrons to the catalytic disulfide of another hydroperoxide reductase, AhpC. In contrast to *E. coli* TrxR, reduced AhpF may form 540 nm absorbing charge-transfer complex under high ionic strength of the solution. AhpF from *E. coli* reduces nitrofurans in a single-electron way [237]. Although the kinetic parameters of these reactions are not reported, AhpH is partly responsible for the sensitivity of *E. coli* to nitrofurans.

Summing up, the low nitroreductase activity of disulfide reductases may be attributed to the low electron density on FAD in FAD-thiolate charge-transfer complexes. This diminishes their role in redox cycling or other modes of activation of ArNO_2_ in the cell. However, low-*M*_r_ TrxRs where FAD and catalytic disulfide function separately may more significantly contribute to the redox cycling of ArNO_2_. The frequently observed single-electron reduction in nitroaromatics is not inconsistent with the redox properties of FAD of disulfide reductases. Apart from the formation of FADH· of low-*M*_r_ TrxR of *E. coli* under the artificial conditions [227], the complex of reduced GR with NADP^+^ also gradually converts into a product, characterized as the complex of NADP^+^ with FAD^−^·([184], and references therein). Another relevant problem is the exploitation of the bioreductive potential of a reduced selenylsulfide moiety of mammalian TrxR in nitroreductase reactions.

## 4. Role of Enzymatic Reduction in Nitroaromatic Compounds in Their Cytotoxicity/Therapeutic Action

### 4.1. Role of Bioreductive Processes in Mammalian Cell Cytotoxicity of Nitroaromatics

Redox cycling is an intrinsic property of ArNO_2_; therefore, the oxidative stress will obligatorily take place during their action under aerobic conditions. In our opinion, the deviation from the limits predicted by the redox cycling activity could be instrumental in the characterization of additional mechanisms of cytotoxicity or therapeutic action of nitroaromatics. It is commonly accepted that if the main cytotoxicity factor is the rate of enzymatic formation of ArNO_2_^−^· and their redox cycling, their cytotoxicity may be described by a following quantitative structure-activity relationship (QSAR):

log cL_50_ = *a* + *b E*^1^_7_ + *c* log *P* (log *D*), (8)

where cL_50_ is compound concentration causing 50% cell death or analogous quantitative parameter, log *P* is octanol/water partition coefficient, and log *D* is octanol/water distribution coefficient at pH 7.0 [13,155,238,239,240,241] (Table 4). These dependences mirror the log (rate constant) vs. *E*^1^_7_ relationships in single-electron reduction in ArNO_2_ by P-450R, NOS, or other single-electron transferring flavoenzymes (see Section 3.1). An additional diagnostic test is antioxidant protection against cytotoxicity. However, this approach may not be applied in the assessment of the mechanisms of action of ArNO_2_ that possess alkylating, bioreductively alkylating, or leaving groups (see below), or may directly interact with DNA, e.g., the positively charged nitracrine (**49**) derivatives [242]. Nevertheless, this approach, in particular, the use of *E*^1^_7(calc.)_ obtained from enzymatic single-electron reduction reactions (Table A1, Appendix A), enabled to demonstrate that the main cytotoxicity mechanism of explosives pentryl (**1**), tetryl (**2**), TNT (**4**), tetranitrobenzimidazolone (**42**), dinitrobenzofuroxane (**48**), ANTA (**54**), NTO (**55**), and nitroaromatic antiandrogens nilutamide (**15**) and flutamide (**16**) could be oxidative stress [13,71,155,241,243,244,245,246]. On the other hand, although nifurtimox (**27**) induces oxidative stress in neuroblastoma cells and is synergistic with the GSH synthesis inhibitor buthionine sulfoximine, it is unclear whether this is the main mechanism of its anticancer action [247,248].

**Table 4:** Structure-activity relationships in mammalian cell cytotoxicity of nitroaromatics with possible relevance to bioreductive activation.

Compounds and Cells	Structure-Activity Relationships
**Aerobic Conditions**	
Nitroimidazoles, nitrofurans, nitrobenzenes, Chinese hamster V79–379A cells [238,239]; nitrobenzenes, nitrofurans, nitrobenzimidazoles, bovine leukemia virus-transformed lamb kidney fibroblast FLK cells [144,240,243]; nitrobenzenes, nitrofurans, nitrothiophenes, murine hepatoma MH22a cells, human colon carcinoma HCT116 cells [13,71]	∆log cL_50_/∆*E*^1^_7_ = −8 ÷ −11 V^−1^, ∆log cL_50_/∆log *P*(*D*) = 0 ÷ −0.3; CB-1954 (**14**), SN-23682 (**19**) and their derivatives follow the same correlation [240]
Nitrobenzenes, FLK cells [245], primary mice splenocytes [241]	∆log cL_50_/∆*E*^1^_7_ = −7.5 ÷ 8.5 V^−1^, increased cytotoxicity of amino and hydroxylamino metabolites of TNT (**4**)
Derivatives of nitracrine (**49**), CHO-AA8 cells [242]	The absence of a relationship between *E*^1^_7_ and cytotoxicity
4-(Alkylamino)-5-nitroquinolines, Chinese hamster ovary CHO-AA8 cells [249]	Cytotoxicity roughly increases with the rate of drug-stimulated O_2_ consumption
*N,N*-bis(2-chloroethyl)-*N*-methyl-*N*-(2-nitrobenzyl) ammonium chloride and its derivatives, CHO-AA8 cells [250]	Alkylating bioreductively activated leaving group does not affect the cytotoxicity, the relationship between *E*^1^_7_ and cytotoxicity is absent
Derivatives of NitroCBI (**54**), Skov3 and HT29 cells [23]	The relationship between *E*^1^_7_ and cytotoxicity is absent
Nitrofurans, nitrothiophenes, nitropyrroles and nitropyrazoles with alkyl-*N*-aziridine or alkyloxirane side chains, V79–379A cells [251,252,253]	Alkylating side chains increase the cytotoxicity, the relationship between *E*^1^_7_ and cytotoxicity is absent
**Hypoxic Conditions**	
Nitroimidazoles, nitrofurans, V79–379A cells [254]	∆log cL_50_/∆*E*^1^_7_ = −10 V^−1^
4-(Alkylamino)-5-nitroquinolines, CHO-AA8 cells [249]	Cytotoxicity and hypoxic selectivity roughly increase with the rate of drug-stimulated O_2_ consumption
Nitrofurans and nitrothiophenes with alkyl-*N*-aziridine or alkyloxirane side chains, V79–379A cells [251,252,253]	Alkylating side chains do not increase hypoxic selectivity
*N,N*-bis(2-chloroethyl)-*N*-methyl-*N*-(2-nitrobenzyl) ammonium chloride and its derivatives, CHO-AA8 cells [250]	Alkylating leaving groups increase cytotoxicity, no relationship between *E*^1^_7_ and cytotoxicity or hypoxic selectivity
Nitrochloromethylbenzindolines (nitroCBIs, **56**), Skov3 and HT29 cells [23]	Hypoxic toxicity does not correlate with *E*^1^_7_, hypoxic selectivity roughly increases with *E*^1^_7_

In this context, one may note that because of the similarity of log (rate constant) vs. *E*^1^_7_ relationships in the reduction in ArNO_2_ by flavoenzymes dehydrogenases-electron-transferases, it is difficult to assess their individual contribution. Some information may be obtained from the hypoxic cytotoxicity studies (see below), assuming that the extent of formation of reduced ArNO_2_ metabolites is proportional to the rate of their single-electron enzymatic reduction. In this case, the main enzyme responsible for redox cycling of ArNO_2_ should be NADPH:cytochrome P-450 reductase (P-450R) [255,256]. The roles of other P-450R-type enzymes such as inducible NO synthase, human novel oxidoreductase NR1 [257], and methionine synthase reductase [258] depend on the cell type. Although the nitroreductase reactions of the latter two enzymes are uncharacterized, one may expect the similarity of their log (rate constant) vs. *E*^1^_7_ relationships with those of P-450R and NOS. On the other hand, the contribution of mitochondrial NADH:ubiquinone dehydrogenase and, possibly, other flavoenzymes of mitochondrial respiratory chain to the cytotoxicity of ArNO_2_ was recently demonstrated [259]. The comparison of reactivities of P-450R and NOS with those of disulfide reductases (Table 1 and Table 2 and Section 3.3) clearly rules out the latter enzymes as the important sources of free radicals of ArNO_2_. The inhibitors of cytochromes P-450 protect against the cytotoxicity of nitrobenzenes, nitrofurans, and nitrothiophenes [71,205] in several cell lines; however, the roles of cytochromes P-450 in ArNO_2_ cytotoxicity remain unclear so far.

The presence of directly alkylating alkyl-*N*-aziridine or alkyloxirane groups in ArNO_2_ molecules evidently increases their cytotoxicity and abrogates the log (cytotoxicity) vs. *E*^1^_7_ relationships (Table 4). It is important to distinguish the contribution of redox cycling and alkylation in their action, in particular, the role of putative alkylation targets. It is suggested that 2-phenylsulfonylnitropyridines, which possess micromolar cL_50_ levels against the NCI cancer cell lines, exert their action through thioredoxin reductase inactivation [219]. However, because of the high *E*^1^_7_ value of nitropyridine moiety (Table A1), the redox cycling reactions with flavoenzymes electrontransferases may significantly contribute to their cytotoxicity. The influence of bioreductively activated alkylating groups such as aziridine of nitrogen mustard or bioreductively activated leaving tetraalkylammonium group in the aerobic cytotoxicity of ArNO_2_ remains equivocal (Table 4). In these cases, an increase in cytotoxicity of ArNO_2_ may be attributed to the formation of a fraction of stable reduction products in cell compartments with reduced O_2_ tension such as nucleus and mitochondria. In cell lines with 100–200 U/mg NQO1 (determined according to quinone reduction rate), dicoumarol partly protects against the cytotoxicity of ArNO_2_. However, the cytotoxicity of CB-1954, SN-23682, and their derivatives is close to the limits predicted by their *E*^1^_7_ [240], and dicoumarol similarly protects against the cytotoxicity of ArNO_2_ that do not contain aziridine or nitrogen mustard groups [71,243].

ArNO_2_ are considered to be the hypoxia-selective toxins or therapeutic agents because their hydroxylamine reduction products formed under hypoxia may alkylate DNA and other nucleophiles. Frequently, their -NHOH or -NH_2_ metabolites demonstrate even higher cytotoxicity than the parent ArNO_2_ ([23,260,261], and references therein). Additionally, the electron-donating character of these groups increases the reactivity of alkylating aziridine or nitrogen mustard groups and accelerates the dissociation of leaving groups. In certain cases, the hypoxic selectivity, i.e., the ratio of cL_50_ under oxic and hypoxic conditions, may exceed 1000. It was established that NADPH:cytochrome P-450 reductase (P-450R) plays a major role in the formation of reduced metabolites of ArNO_2_ under hypoxia [255,256]. One may expect the correlation of hypoxic toxicity with *E*^1^_7_ analogously with the rate of ArNO_2_ reduction. However, this type of correlation is frequently absent (Table 4). Evidently, this reflects the superposition of two opposite factors, the ease of reduction increasing with *E*^1^_7_ of the parent compound and the alkylating reactivity, decreasing with electron-accepting potency of substituents. The studies of CB-1954, SN-23682, and their reduction product have shown that the cytotoxicity is determined mainly by the alkylating reactivity of reduction products, their stability, and interconversion rate [260].

Nitroreductase-transfected cancer cells are the objects of gene-directed enzyme-prodrug therapy (GDEPT). Most typically, NRs, initially *E. coli* NfsB, activated the alkylating compounds such as CB-1954 and SN-23682 or released a drug molecule bound to ArNO_2_ after its reduction (Scheme 1) [7]. Further, we will address the reductive activation of alkylating ArNO_2_, because in the other case, the cytotoxic outcome mostly depends on the properties of the released drug. Early studies have shown that in the presence of exogenous NfsB and NADH in the cell growth medium, the cytotoxicity of CB-1954, SN-23682, and their derivatives roughly correlated with the rate of their enzymatic reduction [162]. The sensitivity to CB-1954 was shown to correlate closely with the level of nitroreductase expression in the cell ([7], and references therein). CB-1954 demonstrated a substantial bystander effect, i.e., killing of non-transfected cells, shown to be mediated via the cell-permeable hydroxylamine metabolite. Among ArNO_2_ that do not possess bioreductively activated alkylating groups, nitrofurazone, but not misonidazole (**35**) or nitracrine (**50**) showed significant activity in NfsB-transfected cell lines. This may be attributed to its better activity as an enzyme substrate.

However, the further use of NfsB in GDEPT was complicated by the relatively low tolerance of patients to large concentrations of CB-1954, required for its efficient NfsB-catalyzed conversion in the cell ([8,15], and references therein). This has spurred a search for alternative nitroreductase enzymes that are more effective at converting CB-1954 at its low concentrations and for alternative nitroaromatic compounds. The large-scale screening of various NRs pointed to the versatility of NfsA-group nitroreductases, which in general possess higher *k*_cat_/*K*_m_ values of CB-1954 than NfsBs. It is suggested that not a higher *k*_cat_ but a much higher affinity (i.e., much lower *K*_m_) for a given substrate is a defining characteristic of the efficiency of the process [262]. However, the nitroreductase gene expression was unpredictable in transfected HCT-116 cells, and there was little correlation between the CB-1954 sensitivity of those cell lines and the *k*_cat_/*K*_m_ of the purified nitroreductases. Possibly, the long-term stable expression of some nitroreductases is not tolerated in human cell lines. Another potential drawback of the NfsA enzymes is their preference for NADPH, as NADPH is usually present at lower concentrations than NADH in mammalian cells. Currently, efforts are undertaken for the construction of selective mutants of NRs and the improvement of their expression [263,264]. Further studies are performed with new compounds such as PR-104A (**13**)**,** TH-302 (**36**), and nitroCBI (**56**).

Nitroaromatic compounds induce both necrotic and apoptotic mammalian cell death. Thus, finally, one has to address the expression of cell signaling proteins during the apoptotic action of ArNO_2_. The current studies are confined to the changes of expression and phosphorylation of the ‘classical’ signaling proteins such as tumor suppressor p53, cell cycle inhibitor p21, proapoptotic protein Bax, antiapoptotic protein Bcl-2, protooncogene protein N-Myc, various caspases (cysteine-dependent aspartate-directed proteases, which are the executioners of the apoptotic cell death), mitogen-activated kinases JNK, p38 and ERK, and other kinases (Table 5). In certain cases, the upregulation of p21, p53, Bax/Bcl-2 ratio, and caspase expression was attenuated by antioxidants [265,266,267]. This shows that ArNO_2_^−^ initiated oxidative stress contributes to these events.

### 4.2. Role of Bioreductive Processes in Antibacterial and Antiparasitic Action of Nitroaromatics

The current progress in the design and development of nitroaromatic antibacterial and antiparasitic agents was recently presented in several comprehensive reviews [6,268]. In this subsection, we will address the cases with evidence and, preferentially, quantitative demonstration of the contribution of reductive processes to the action of ArNO_2_.

**Table 5:** Expression of cell signaling proteins during the apoptotic action of nitroaromatic compounds.

Cell. Signaling Protein	Effect	Compound, Cells
p53	Upregulation	Furazolidone, human hepatocytes LO2 [265]; phenylnitrobenzene B16, human cervical carcinoma HeLa [269]
	Upregulation, downregulation of mutant p53	Nitrofuran NSC59984, colorectal cancer [270]
	Activation of phosphorylation	1-Nitropyrene, human hepatoma HepG2 [271]
p21	Upregulation	Indolin-2-one-subsituted nitrobenzene HO1-02, esophageal cancer [271]; 1,3-dinitrobenzene, rat Sertoli cells [272]
	Downregulation	Furazolidone, HepG2 [267]
Transcription factor STAT-3	Inhibition of phosphorylation	Nifuroxazide, human myeloma [273]
Bax/Bcl-2 ratio	Upregulation	Phenylnitrobenzene B16, HeLa [269]; 1,3-dinitrobenzene, rat Sertoli cells [272]; furazolidone, HepG2 [267]; *N*-pentyl-furantoin, human promyelocytic leukemia HL-60 [274]; 2-(2-(5-nitrofuran-2-yl)-vinyl)-quinoline derivative, non-small cell lung cancer H1299 [275]; nifuroxazide, human breast cancer MCF-6 [276]
N-Myc	Downregulation	Nifurtimox, neuroblastoma cells [247]
Caspases	Upregulation	Tetryl, HeLa [216]; HO1-02, esophageal cancer [266]; furazolidone, HepG2 [267]; nifuroxazide, MCF-6 [276]; CB-1954, NR-transfected ovarian carcinoma SKOV3 [277]; TNT, HepG2 [278]
JNK	Activation of phosphorylation	1,3-dinitrobenzene, rat Sertoli cells [272]
p38		2-(2-(5-Nitrofuran-2-yl)-vinyl)-quinoline derivative H1299 [275]
ERK	Inhibition of phosphorylation	Nifurtimox, neuroblastoma cells [248]; 5-nitroimidazole DYT-40, human glyoma U251 and U87
	Activation of phosphorylation	1,3-dinitrobenzene, rat Sertoli cells [272]
AKT kinase	Inhibition of phosphorylation	2-(2-(5-Nitrofuran-2-yl)-vinyl)-quinoline derivative, H1299 [275]; 5-nitroimidazole DYT-40, U251 and U87 [279]; nifurtimox, human neuroblastoma [280]

Nitroreductase-deficient mutants of *E. coli* were resistant to all types of nitroaro- matic compounds [281,282]. This points to the principal role of NRs in their bioactivation. Reflecting the correlation between the reactivity of NfsA and NfsB and *E*^1^_7_ of nitroaromatics, the rates of ArNO_2_ aerobic reduction by *E. coli* increased with their electron-accepting potency [283]. However, the activity of ArNO_2_ against *E. coli* and other aerobic bacteria poorly correlated with their electron affinity [284]. This may point to the role(s) of their other insufficiently studied targets such as AhpF [237] or to a parallel activity dependence on the alkylating potency of reduction products, which decreases with the electron-donating potency of substituents [285]. In particular, the activity of a large series of nitroaromatics against anaerobic *Bacteroides fragilis* decreased with their *E*^1^_7_ [284]. On the other hand, there was a reasonable correlation between the *k*_cat_/*K*_m_ of 20 purified NRs with CB-1954 as substrate and their ability to induce CB-1954-mediated DNA damage in *E. coli* host cells [15].

Metronidazole and other 5-nitroimidazoles exert a high antitrichomonal activity under both aerobic and anaerobic conditions; in most experiments, however, inhibitory concentrations are higher in aerobiosis than in anaerobiosis [286]. Trichomonad flagellates and anaerobic bacteria become resistant to metronidazole after a loss of PFOR and NADH:ferredoxin reductase (FOR) functions [120,286]. The unexpectedly high rate of reaction of nitroimidazoles by ferredoxin that initiates redox cycling [110] possibly explains the high sensitivity of *T. vaginalis* to metronidazole. Because the rate of reduction ArNO_2_ by ferredoxin is apparently insensitive to their *E*^1^_7_ [121], it points to the perspectives of application of low-potential compounds that are nontoxic to mammalian cells. However, the further steps are complicated by the uncharacterized nitroreductase reactions of FOR and a parallel involvement of 26 kD FMN and FeS-containing protein with uncharacterized functions [122], which, under anaerobiosis, reduces metronidazole to amino metabolite and contributes to its detoxification. Because of low nitroreductase activity, the role of TrxR in the activation of ArNO_2_ in *T. vaginalis* remains a matter of debate [234,235].

The current studies of *Helicobacter pylori* most frequently address the mechanisms of its resistance to metronidazole [287]. The sensitivity to metronidazole correlated with NADH- and NADPH-dependent nitroreductase activity in the cytosolic fraction of clinical isolates [288]. In turn, resistance to metronidazole results from loss-of-function mutations in genes encoding nitroreductases RdxA and FrxA ([268], and references therein). However, the individual roles of the above NRs in the action of other nitroaromatic compounds remain poorly understood so far. Moreover, another potential candidate for the efficient reduction in ArNO_2_ is the complex of PFOR, FqrB, and flavodoxin [124]. Nitroaromatic compounds that bind to flavodoxin at micromolar concentrations exhibit activity against *H. pylori* at similar concentrations. Importantly, these compounds were active against metronidazole-resistant isolates, which may point to a different mechanism of their action [289]. Further studies in this direction warrant detailed examination of nitroreductase activity of FqrB and, especially, flavodoxin.

Giardiasis is treated primarily with metronidazole, but other ArNO_2_, including benznidazole, nitazoxanide, and nitrofurans, are also effective [125]. *Giardia* spp. possess numerous potential sources of their activation, such as PFOR, ferredoxin, thioredoxin reductase, and several nitroreductases ([125,290,291], and references therein). However, their individual contributions to the action of particular drugs remain a matter of debate.

The recent review [6] did not reveal the relationships between the antitubercular activity of nitroaromatics and their redox properties. The activity of a series of derivatives of PA-824 (**57**) against *M. tuberculosis* did not depend on their *E*^1^_7_ value in the range of −0.570–−0.338 V [24] or their reduction potentials in acetonitrile [292]. On the other hand, 6,8-dinitro-1,3-benzothiazin-4-ones were more active than their 6-trifluoromethyl-8-nitro analogs [293]. Although the downregulation of F-420-dependent nitroreductase confers the resistance of *M. tuberculosis* to PA-824 and other nitroaromatics [294], the studies of several series of PA-824 derivatives did not demonstrate the relationship between their enzymatic reactivity and antibacterial activity [182,295]. In our opinion, the further development of antitubercular nitroaromatics could be extended by studies of their interaction with *M. tuberculosis* flavoenzymes, e.g., FprA [102] or LipDH [208].

Nifurtimox (**27**) and benznidazole (**34**) were the only nitroaromatic compounds to be recently considered for antitrypanosomal drug development ([296], and references the- rein). Because of its genotoxicity, megazol (**38**) was not further developed. The mechanisms of antitrypanosomal action of nifurtimox and other nitrofurans are a matter of debate. The early studies suggested that the main mechanism of action is their single-electron reduction and redox cycling of free radicals, the potential candidates being trypanothione reductase, lipoamide dehydrogenase, and, possibly, P-450R-type enzymes [199,203,297]. However, redox cycling was only observed at high nifurtimox concentrations, which were by two orders of magnitude higher than its micromolar concentrations required for antiproliferative activity [297]. On the other hand, the two-electron transferring trypanosomal NTRs may play a leading role in the bioactivation of nifurtimox and, possibly, other nitrofurans and nitroimidazoles [174,175,176]. Their upregulation or downregulation increased the sensitivity or resistance of parasites, respectively, toward nifurtimox, benznidazole, and fexinidazole. However, because of limited kinetic data on NTR reactions, including the new groups of ArNO_2_ such as 3-nitrotriazoles and nitroquinolin-2(1*H*)-ones [298,299,300,301,302], it is difficult to demonstrate the relationship between their trypanocidal activity and reactivity toward NTR. Moreover, in certain cases, the upregulation or downregulation of NTR causes limited effects on parasite sensitivity. In this context, parallel studies of other flavoenzyme targets of nitroaromatics may be suggested. For example, the nitroreductase activity of trypanothione reductase (TR) is similar to that of NTRs [199,200], although the reaction proceeds in a single-electron way. Moreover, nitrofurans act as TR inhibitors [199,200]. There did not exist a relationship between the trypanocidal activity and TR inhibition potency of a series of nitrofurans, which were weak enzyme inhibitors (*K*_i_ = 61–1180 µM) [303]. However, the trypanocidal activity of chinifur (**24**) and its derivatives which were more efficient TR inhibitors (*K*_i_ = 2.3–110 µM), increased with their TR inhibition potency [304].

NR1 of *Leishmania* spp. participates in the activation of the new antiparasitic agent fexinidazole (**38**) and nifurtimox (**27**), because its upregulation increases the parasite sensitivity to these drugs. On the other hand, the knockout of NR2 caused the parasite complete resistance *R*-PA-824 (**57**) and only marginal resistance to nifurtimox [179,296]. This is in line with their relative efficiency as NR2 substrates.

The action of ArNO_2_ against the malaria parasite *P. falciparum* received relatively little attention, although a number of nitrofurans, nitrobenzenes, nitroimidazoles, and 4-nitrobenzothiadiazole were shown to possess in vitro antiplasmodial activity at micromolar or lower concentrations [17,195,305,306]. The antiplasmodial activity of a series of nitrobenzenes and nitrofurans increases with their *E*^1^_7_, log *D*, and their efficiency of inhibition of *P. falciparum* glutathione reductase (*Pf*GR) [195]. The relationship between the activity of ArNO_2_ and the inhibition of erythrocyte GR is uncertain. Given the data available, *P. falciparum* ferredoxin:NADP^+^ oxidoreductase (*Pf*FNR), and, possibly, ferredoxin may act as the most efficient reductants of ArNO_2_ in plasmodia. *P. falciparum* thioredoxin reductase may be next to it according to quinone reductase activity [221]. Because *P. falciparum*, during its intraerythrocyte stage, adopts microaerophilic metabolism and relies mainly on anaerobic processes [307], an increase in the activity of ArNO_2_ with *E*^1^_7_ may equally well reflect the rates of formation of free radicals or DNA-damaging hydroxylamines under the action of *Pf*FNR. On the other hand, the series of tetrazole-containing nitroimidazooxazines and quinoline-based nitroimidazoles exhibited antiplasmodial activity at micromolar concentrations [306], which is beyond the limits expected from their redox potentials (Table A1). This may be attributed to additional modes of their action arising from the hybrid structure of these compounds.

## 5. Conclusions

This review is not meant to be exhaustive in addressing the numerous and complex ways of interaction of nitroaromatic compounds with flavoenzymes and their cytotoxic/therapeutic consequences. However, one may conclude that with some minor exceptions, the single-electron reduction potential of nitroaromatics is the principal factor determining the rate of their single-electron reduction by flavoenzymes dehydrogenases-electrontransferases and their FeS redox partners. This leaves relatively little space for the improvement of the enzymatic reactivity of compounds. On the other hand, although the studies of several two-electron transferring bacterial nitroreductases point to an important role of redox potential in the reactivity of nitroaromatics, the factors determining substrate specificity of nitroreductases from *H. pylori*, *H. influenza*, *Leishmania,* and *Trypanosoma* spp. remain unclear. This leaves more space for the improvement of the activity of compounds.

Low nitroreductase activity of flavoenzymes disulfide reductases diminishes their role in redox cycling or other modes of activation of nitroaromatics in the cell. However, low-*M*_r_ TrxRs where FAD and catalytic disulfide function separately may more significantly contribute to the redox cycling of nitroaromatics. Another relevant problem is the exploitation of the bioreductive potential of a reduced selenylsulfide moiety of mammalian TrxR. In our opinion, this review may highlight the potential relevance of a number of flavoenzymes and/or their metalloprotein redox partners whose nitroreductase reactions were studied less comprehensively. Because of the definite perspectives of nitroaromatic compounds in the treatment of oxic tumors, the potential but insufficiently characterized targets of their action are the mitochondrial respiratory chain, in particular, its complex I and cytochromes P-450. Concerning the flavoenzymes of bacteria and parasites, more thorough studies of nitroreductase reactions of FprA and LipDH of *M. tuberculosis*, flavodoxin of *H. pylori*, TrxR, and Fd of *P. falciparum*, and PFOR/Fd/FOR systems of *Trichomonas* and *Giardia* spp. may foster the development of new groups of nitroaromatic agents or the repurposing of existing ones.

## Figures and Tables

**Table 1:** Potentiometric and kinetic characteristics of heme-reducing flavoenzymes and flavohemoproteins.

Enzyme	Redox Potential vs. NHE	Rate Constants of Electron (Hydride) Transfer, pH 7.0
NADPH: cytochrome P-450 reductase (rat liver)	−0.325 V (FAD/FADH·), −0.372 V (FADH·/FADH_2_), −0.068 V (FMN/FMNH·), −0.246 V (FMNH·/FMNH_2_), pH 7.4 [60]	30 s^−1^ (NADPH to cytochrome *c* via FAD and FMN, steady-state) [61], *k*_cat_/*K*_m_ = 6.8 × 10^2^–5.9 × 10^6^ M^−1^s^−1^ (ArNO_2_, steady-state) [13]
Nitric oxide synthase (rat neurons)	−0.250 V (FAD/FADH·), −0.260 V (FADH·/FADH_2_), −0.120 V (FMN/FMNH·), −0.220 V (FMNH·/FMNH_2_), −0.290 V (Fe^3+^/Fe^2+^), H 7.0 [62]	242 s^−1^ (NADPH to flavins, fast phase), 46 s^−1^ (FMNH_2_ to heme), pH 7.6 [63]; *k*_cat_/*K*_m_ = 1.2 × 10^2^–2.8 × 10^5^ M^−1^s^−1^, (steady-state, ArNO_2_, calmodulin is absent) [64]
Flavohemoglobin (bacteria, fungi)	≤−0.150 V (FAD/FADH^−^), −0.120 V (heme) (*E. coli* FHb, pH 8.0) [65]; −0.190 V (FAD/FADH^−^), −0.170 V (heme) (*S. aureus* FHb, pH 7.6) [66]	130 s^−1^ (NADH to heme via FAD), *k*_cat_ = 4–25 s^−1^, *k*_cat_/*K*_m_ = 6.2 × 10^2^–1.1 × 10^5^ M^−1^s^−1^ (steady-state, ArNO_2_, *S. aureus* FHb) [67]

**Table 2:** Potentiometric and kinetic characteristics of flavoenzymes FeS reductases and FeS proteins.

Enzyme	Redox Potential vs. NHE, pH 7.0	Rate Constants of Electron (Hydride) Transfer, pH 7.0
Bovine adrenal cortex NADPH: adrenodoxin reductase	−0.250–−0.274 V (FAD/FADH_2_), −0.320 V (FAD/FADH·), −0.260 V (ADX, free), −0.360 V (ADX, ADR-bound) [86,87,88]	28 s^−1^ (NADPH to FAD), 10 e^−^/s (FADH_2_ to ADX_ox_,steady-state) [86,87,88]
Ferredoxin: NADP(H) oxidoreductase, *Anabaena* PCC7119 [89,90,91]	−0.296 V (FAD/FADH^−^), −0.280 V (FAD/FADH·), −0.384 V(Fd), pH 7.5	6200 e^−^/s (Fd_red_ to FAD), 250 e^−^/s (Fd_red_ to FADH), >600 s^−1^ (FADH^−^ to NADP^+^), pH 8.0; 140 s^−1^ (NADPH to FAD), >600 s^−1^ (FADH^−^ to Fd_ox_)
Ferredoxin: NADP(H) oxidoreductase, *Plasmodium* *falciparum* [92,93]	−0.280 V (FAD/FADH^−^), −0.260 V (Fd)	125–148 s^−1^ (NADPH to FAD) 13–15 e^−^/s (FADH^−^ to Fd_ox_, steady-state)
Bovine heart mitochondrial NADH:ubiquinone reductase (complex I)	−0.345 V (FMN/FMNH_2_), −0.382 V (FMN/FMNH·) [94]; ≤−0.380 V (N1a), −0.240–−0.270 V (N1b, N3, N4, N5), −0.050–−0.120 V (N2) [95]	3500 e^−^/s (NADH to FMN (ferricyanide), steady-state) [96,97]; 150–380 s^−1^ (NADH to ubiquinone, steady-state, proteoliposomes) [98]

## Appendix A

**Table A1:** Single-electron reduction midpoint potentials of nitroaromatic compounds determined by pulse radiolysis (*E*^1^_7_) or calculated according to the data of enzymatic single-electron reduction (*E*^1^_7(calc.)_).

No.	Compound	*E*^1^_7_ (V) ^a^	*E*^1^_7(calc.)_ (V) ^b^
	**Nitrobenzenes**		
**1**	2,4,6-Trinitrophenyl-*N*-nitramino-ethylnitrate (pentryl)	-	−0.175
**2**	2,4,6-Trinitrophenyl-*N*-methylnitramine (tetryl)	-	−0.191
**3**	*N*-Methylpicramide	-	−0.247
**4**	2,4,6-Trinitrotoluene (TNT)	−0.253 ^c^	−0.279
**5**	1,4-Dinitrobenzene	−0.257	−0.240
**6**	1,2-Dinitrobenzene	−0.287	−0.311
**7**	3,5-Dinitrobenzamide	-	−0.311
**8**	4-Nitrobenzaldehyde	−0.325	−0.342
**9**	3,5-Dinitrobenzoic acid	−0.344	−0.336
**10**	1,3-Dinitrobenzene	−0.348	−0.332
**11**	2-Hydroxylamino-4,6-dinitrotoluene	-	−0.351
**12**	4-Nitroacetophenone	−0.355	−0.356
**13**	2-((2-Bromoethyl)(2,4-dinitro-6-((2-(phosphonooxy)ethyl)carbamoyl)phenyl)amino)ethyl methanesulfonate (PR-104A)	−0.366 ^d^	
**14**	5-(Aziridin-1-yl)-2,4-dinitrobenzamide (CB-1954)	−0.385	−0.352
**15**	5,5-Dimethyl-3-(4-nitro-3-(trifluoromethyl)phenyl) imidazoline-2,4-dione (nilutamide)	-	−0.399
**16**	2-Methyl-*N*-(4-nitro-3-(trifluoromethyl)phenyl) propanamide (flutamide)	-	−0.408
**17**	2-Amino-4,6-dinitrotoluene	−0.417 ^c^	−0.423
**18**	4-Nitrobenzoic acid	−0.425	−0.423
**19**	5-(Bis(2,2′-chloroethyl)amino)-2,4-dinitrobenzamide (SN-23682)	−0.425	−0.398
**20**	4-Hydroxylamino-2,6-dinitrotoluene	-	−0.429
**21**	4-Amino-2,6-dinitrotoluene	−0.449 ^c^	−0.453
**22**	Nitrobenzene	−0.485	−0.489
**23**	*D*-(-)-threo-2-dichloroacetamido-1-(4-nitrophenyl)-1,3- propandiol (chloramphenicol)	−0.546 ^e^	
**Nitrofurans and nitrothiophenes**			
**24**	2-(5′-Nitrofurylvinyl)-quinoline-4-carboxydiethylamino- 1-methyl-but-1-ylamide (chinifur, quinifuryl)	−0.225 ^f^	−0.221
**25**	5-Nitro-2-furaldoxime (nifuroxime)	−0.255	−0.279
**26**	*N*-(5-nitro-2-furfurylindene)-1-aminohydantoin (nitrofurantoin)	−0.265	−0.263
**27**	4-(5-Nitrofurfurylidenamino)-3-methyltiomorpholine- 1,1-dioxide (nifurtimox)	−0.260 ^g^	−0.285
**28**	2-Nitrothiophene-5-aldehyde	−0.260 ^h^	
**29**	2-Nitrothiophene-5-aldoxime	−0.280 ^h^	
**30**	2-Nitrothiophene-5-carboxymorpholide	−0.305 ^h^	
**31**	2-Nitrofuran	−0.330	
**32**	2-Nitrothiophene	−0.390	
**Nitroimidazoles**			
**33**	1-Methyl-2-nitroimidazole-5-carboxamide	−0.321	
**34**	*N*-Benzyl-2-(2-nitro-1*H*-imidazol-1-yl)acetamide (benznidazole)	−0.380	
**35**	1-Methoxy-3-(2-nitroimidazol-1-yl)propan-2-ol (misonidazole)	−0.389	
**36**	1-(Methyl-2-nitro-1*H*-imidazole-5-yl)-methyl-*N,N*’-bis(2- bromoethyl)phosphorodiamidate (TH-302, evofosfamide)	−0.407 ^i^	
**37**	2-Nitroimidazole	−0.418	
**38**	1-Methyl-2-(5-amino-1,3,4-thiadazole)- 5-nitroimidazole (megazol)	−0.438 ^g^	
**39**	1-Methyl-2-((4-(methylthio)phenoxy)methyl)-5-nitro-1H- imidazole (fexinidazole)		−0.458
**40**	2-Methyl-5-nitroimidazole-1-ethanol (metronidazole)	−0.486	
**41**	4-Nitroimidazole	≤−0.527	
**Nitrobenzimidazoles**			
**42**	4,5,6,7-Tetranitrobenzimidazolone	-	−0.197
**43**	4,5,6-Trinitrobenzimidazolone	-	−0.224
**44**	5,6-Dinitrobenzimidazolone	-	−0.273
**45**	2-Nitrobenzimidazole	−0.300	-
**46**	5-Nitrobenzimidazolone	-	−0.355
**Miscellaneous**			
**47**	4-Nitropyridine	−0.190	
**48**	4,6-Dinitrobenzofuroxane	-	−0.258
**49**	5-Nitro-4-(3′-dimethylaminopropylamino)-quinoline (nitraquine)	−0.286 ^j^	
**50**	1-Nitro-9-(3′-dimethylaminopropylamino)-acridine (nitracrine)	−0.303 ^j^	−0.285
**51**	1-*N*-alkyl-3-nitro-1,2,4-triazines	−0.310–−0.330 ^k^	
**52**	2-((5-Nitrothiazol-2-yl)carbamoyl)phenyl acetate (nitazoxanide)		−0.380
**53**	5-Nitrothiazole	−0.400	
**54**	5-Amino-3-nitro-1,2,4-triazole (ANTA)	-	−0.466
**55**	3-Nitro-1,2,4-triazolone (NTO)	-	−0.472
**56**	1-(Chloromethyl)-3-(5-(2-(dimethylamino)-ethoxy)indol- 2-carbonyl)-5-nitro-1,2-dihydro-3*H*-benzo[*e*]-indole (representative of nitroCBIs)	−0.512 ^l^	
**57**	2-Nitro-6((4-(trifluoromethoxy)benzyl)oxy)-6,7- dihydro-5*H*-imidazo [2,1-*b*][1,3]oxazine (PA-824)	−0.534 ^m^	

^a^ Taken from Ref. [10] unless specified otherwise, ^b^ taken from Refs. [11,12,13], ^c^ taken from Ref. [14], ^d^ taken from Ref. [15], ^e^ taken from Ref. [16], ^f^ taken from Ref. [17], ^g^ taken from Ref. [18], ^h^ taken from Ref. [19], ^I^ taken from Ref. [20], ^j^ taken from Ref. [21], ^k^ taken from Ref. [22], ^l^ taken from Ref. [23], ^m^ taken from Ref. [24].

## Appendix B

The single-electron reduction in nitroaromatics is frequently treated according to an “outer-sphere” electron transfer model, which describes an electron transfer with weak electronic coupling between the reactants ([28], and references therein). In the simplest form, the rate constants of single-electron transfer between reagents (*k*_12_) depend on the electron self-exchange rate constants of reagents (*k*_11_ and *k*_22_) and equilibrium constant of reaction (*K*) (log *K* = ∆*E*^1^ (V)/0.059, where ∆*E*^1^ is the difference in the standard single-electron transfer potentials of reactants):

*k*_12_ = (*k*_11_ × *k*_22_ × *K* × *f*)^1/2^ (A1)

and

log *f* = (log *K*)^2^/4log (*k*_11_ × *k*_22_/*Z*^2^), (A2)

where *Z* is a frequency factor (10^11^ M^−1^s^−1^). According to Equations (A1) and (A2), in the reaction of electron donor with a series of homologous electron acceptors (*k*_22_ = constant), *k*_12_ will exhibit parabolic (square) dependence on ∆*E*^1^ with a slope ∆log *k/*∆∆*E*^1^ = 8.45 V^−1^ at ∆*E*^1^ = ±0.15 V. At ∆*E*^1^ = 0, *k*_12_ = (*k*_11_ × *k*_22_)^1/2^. For ArNO_2_, the self-exchange constants are around 10^6^ M^−1^s^−1^ [25,26]. For quinones and free flavins, they are around 10^8^ M^−1^s^−1^ ([29], and references therein). In the reactions of redox proteins, the protein *k*_11_ values may be used to evaluate the orientational distances of electron transfer (*R*_p_) [30]:

*R*_p_ (Å) = 6.3 − 0.35 ln *k*_11_. (A3)
